# Indole Derivatives Bearing Imidazole, Benzothiazole-2-Thione or Benzoxazole-2-Thione Moieties—Synthesis, Structure and Evaluation of Their Cytoprotective, Antioxidant, Antibacterial and Fungicidal Activities

**DOI:** 10.3390/molecules28020708

**Published:** 2023-01-10

**Authors:** Beata Jasiewicz, Karolina Babijczuk, Beata Warżajtis, Urszula Rychlewska, Justyna Starzyk, Grzegorz Cofta, Lucyna Mrówczyńska

**Affiliations:** 1 Department of Bioactive Products, Faculty of Chemistry, Adam Mickiewicz University, Uniwersytetu Poznańskiego 8, 61-614 Poznań, Poland; 2 Department of Crystallography, Faculty of Chemistry, Adam Mickiewicz University, Uniwersytetu Poznańskiego 8, 61-614 Poznań, Poland; 3 Department of Soil Science and Microbiology, Faculty of Agronomy, Horticulture, and Bioengineering, University of Life Science, Szydłowska 50, 60-656 Poznań, Poland; 4 Department of Wood Chemical Technology, Faculty of Forest and Wood Technology, University of Life Science, Wojska Polskiego 28, 60-637 Poznań, Poland; 5 Department of Cell Biology, Faculty of Biology, Adam Mickiewicz University, Uniwersytetu Poznańskiego 6, 61-614 Poznań, Poland

**Keywords:** gramine, indole derivatives, antioxidant properties, antibacterial activity, fungicidal properties, chalcogen replacement, S/O equivalence

## Abstract

In the search for new bioactive compounds, a methodology based on combining two molecules with biological properties into a new hybrid molecule was used to design and synthesize of a series of ten indole derivatives bearing imidazole, benzothiazole-2-thione, or benzoxazole-2-thione moieties at the C-3 position. The compounds were spectroscopically characterized and tested for their antioxidant, antibacterial, and fungicidal activities. The crystal structures were determined for five of them. Comparison of the closely related structures containing either benzothiazole-2-thione or benzoxazole-2-thione clearly shows that the replacement of -S- and -O- ring atoms modify molecular conformation in the crystal, changes intermolecular interactions, and has a severe impact on biological activity. The results indicate that indole-imidazole derivatives with alkyl substituent exhibit an excellent cytoprotective effect against AAPH-induced oxidative hemolysis and act as effective ferrous ion chelating agents. The indole-imidazole compound with chlorine atoms inhibited the growth of fungal strains: *Coriolus versicolor (Cv), Poria placenta (Pp), Coniophora puteana (Cp),* and *Gloeophyllum trabeum (Gt)*. The indole-imidazole derivatives showed the highest antibacterial activity, for which the largest growth-inhibition zones were noted in *M. luteus* and *P. fluorescens* cultures. The obtained results may be helpful in the development of selective indole derivatives as effective antioxidants and/or antimicrobial agents.

## 1. Introduction

The synthesis of compounds based on nitrogen-, oxygen-, and sulfur-containing heterocyclic scaffolds has become increasingly important in recent years, particularly in medical chemistry [1]. Among nitrogen-heterocyclic compounds, indole derivatives deserve special attention due to their broad spectrum of biological activity. Synthetic indoles are promising therapeutic agents and have been found to exhibit antiviral, antibacterial, anti-inflammatory, and antidepressant activities [2,3,4,5]. Many of them have been approved as drugs, such as indomethacin, pindolol, and indapamide [6]. The indole structure is also present in the indole-3-carbinol, an important antitumor agent [7].

The indole moiety is widely distributed in nature. Naturally derived indole compounds include, among others, the amino acid tryptophan, tryptamine, serotonin, and melatonin [8]. Additionally, tryptophan is a precursor of several indole alkaloids, including reserpine, vincristine, yohimbine, and gramine [9,10,11,12,13].

One of the most important biological activities of indole derivatives is their antioxidant potential. The antioxidant properties of melatonin are probably due to the presence of the C-3 ethylamido side chain [14]. Also, synthetic indoles, bearing substitutes at the C-3 positions, are promising antioxidant agents [14,15,16,17]. Our previous study showed that gramine substituted by the pyrrolidinedithiocarbamate moiety has significant antioxidant properties [18]. Moreover, selected C-3 substituted indole-uracils [19] and indole-triazole [20] conjugates were able to inhibit AAPH-induced oxidative hemolysis effectively.

Another important class of nitrogen-containing heterocycles is imidazoles. The imidazole ring system is found in the amino acid histidine, hormone histamine, and purine [21]. Imidazole derivatives have antibacterial, anticancer, antitubercular, antifungal, and analgesic properties [22,23]. 

Benzothiazole and its related heterocycle benzoxazole are other versatile scaffolds in organic chemistry [24,25]. Their derivatives possess various biological activities, including anti-inflammatory, antitubercular, antifungal, and anti-diabetic properties [25,26,27,28]. Recently, Karaca et al. showed that some benzothiazole derivatives have a promising potential for the treatment Alzheimer’s and Parkinson’s diseases [29]. 

One of the methods for designing novel drug candidates is to combine two molecules with biological properties into a new hybrid with better affinity compared to the individual parent compounds [30]. 

**Figure 1:**
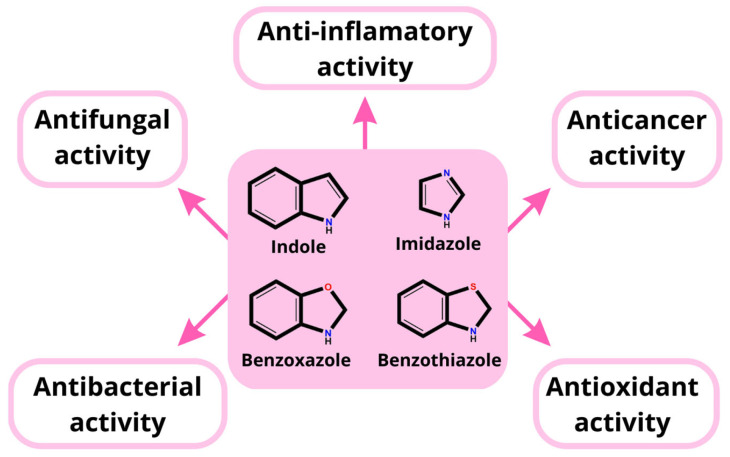
Organic chemistry scaffolds.

As part of our ongoing research into the design of novel bioactive C-3 substituted indole derivatives and considering the importance of heterocyclic skeletons for developing bioactive compounds, we synthesized and characterized indole-imidazole, indole- benzothiazole-2-thione, or indole-benzoxazole-2-thione hybrids in an attempt to design effective antioxidant agents. Structural heterocyclic scaffolds used for synthesizing the target compounds are shown in Figure 1.

The synthesized hybrids were evaluated for their ability to complex ferrous ions and inhibitory effect on 2,2′-azobis- (2-amidinopropane hydrochloride) (AAPH)–induced hemolysis of human erythrocytes. 

In addition, the compounds obtained were screened for possible antifungal and antibacterial activities. 

## 2. Results and Discussion

### 2.1. Synthesis and Spectroscopic Characterization of C-3 Substituted Indole Derivatives

**Figure 2:**
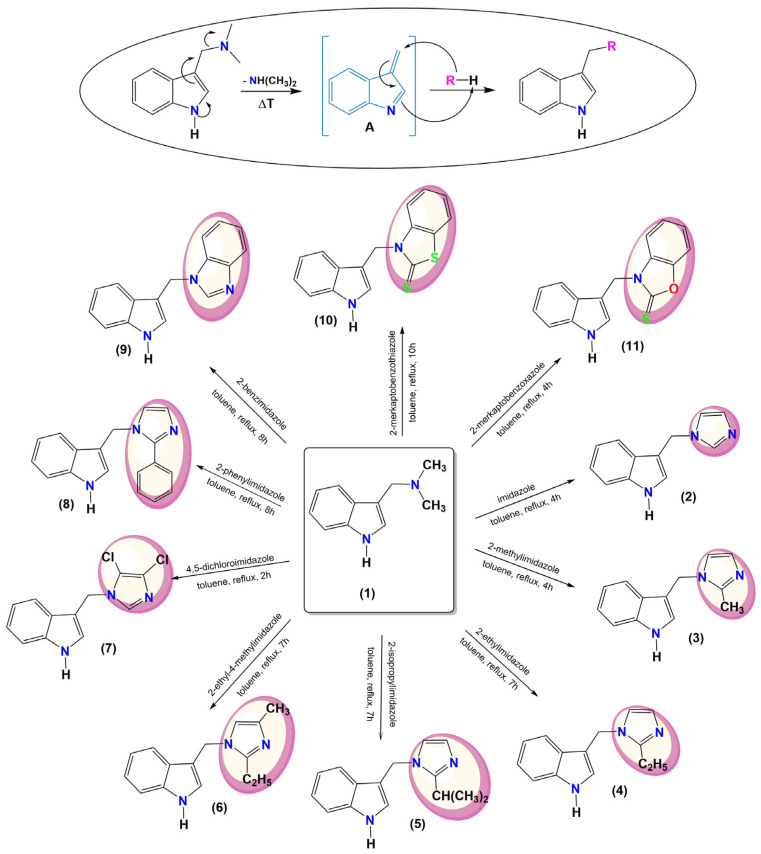
Synthesis of indole derivatives with imidazole, benzothiazole–2–-thione and benzoxazole–2–thione moieties.

To obtain C-3 substituted indole derivatives, gramine was used as a substrate with a good leaving group leading to a vinylogous intermediate [A]. The reaction of [A] with appropriate imidazole compounds (imidazole, 2-methylimidazole, 2-ethylimidazole, 2-isopropylimidazole, 2-ethyl-4-methylimidazole, 2,5-dichloroimidazole, 2-phenylimidazole, 2-benzimidazole), 2-mercaptobenzothiazole or 2-mercaptobenzoxazole gives compounds **2**–**11** (Figure 2).

Indole-imidazole compounds are known in the literature [31,32,33,34,35,36,37,38,39], but some proposed syntheses are multi-step or require special reaction conditions [36,37,38]. Our one-pot synthesis method is based on a compound of natural origin, is simple, and does not need column chromatography.

The structures of all obtained compounds were determined by analyzing their ^1^H and ^13^C NMR, FT-IR, and EI-MS spectra. The spectroscopic data of literature-known compounds **2** and **9** agreed with those found in the references [18,38]. ^13^C NMR spectra of compounds **2**–**11** showed signals in the range of 108–136 ppm corresponding to indole moiety and near 180 ppm indicating the presence of a thiocarbonyl group (compounds **10** and **11**). Characteristic signals at about 118–152 ppm (compounds **2**–**9**) were assigned to the imidazole ring and at 110–146 ppm to benzimidazole, benzothiazole-2-thione, and benzoxazole-2-thione moiety (compounds **9**–**11**). Additionally, signals of alkyl (methyl, ethyl, and isopropyl) groups for compounds **3**–**6** were observed in the 12–25 ppm range. The ^1^H NMR spectra of compounds **2**–**11** showed characteristic hydrogen singlets near 11 ppm that were assigned to the NH protons of gramine moiety and signals in the range 6.68–7.69 ppm for protons of the indole fragment. The protons of the C(10)H_2_ group gave signals near 5.00 ppm, and protons of the imidazole ring at 6.69–7.37 ppm. The protons of the alkyl groups (compounds **3**–**6**) gave signals at 1.95–2.33, 1.12–2.69, and 1.15–3.29 ppm for methyl, ethyl, and isopropyl group, respectively. Characteristic signals of benzimidazole, benzothiazole-2-thione, and benzoxazole-2-thione protons are at 7.15–7.83 ppm. The NMR spectra (^1^H and ^13^C) of the investigated compounds are provided in the Supplementary part (Figures S1–S9). The FT-IR spectra of all compounds in KBr tablets showed the characteristic band at 3550–3300 cm^−1^ derived from the NH of the indole ring and characteristic indole ring absorption at 650–540 cm^−1^. Moreover, in the spectra of 10 and 11, there are narrow absorption bands from the S=C bond at 1266 and 1285 cm^−1^, respectively. The aromatic character of compounds was confirmed by the presence of absorption bands at 1625–1575 cm^−1^ and 1525–1450 cm^−1^. In the EI-MS spectra of almost all compounds (except **8**), molecular ions were observed, and their relative abundance was 4–74%. For all new derivatives, the ion with an intensity of 100% is at *m/z =* 130 (C_9_H_8_N^+^). The NMR (^1^H and ^13^C), EI-MS, and FT-IR spectra of the investigated compounds are provided in the Supplementary Materials (Figures S1–S27).

### 2.2. X-Ray Analysis

Single crystals suitable for X-ray diffraction were obtained for six derivatives (**4**, **5**, **8**, **9**, **10**, **11**). Since the crystal structure of **9** has already been determined [38], we report here the results of a single crystal X-ray analysis for the other five compounds. 

**Figure 3:**
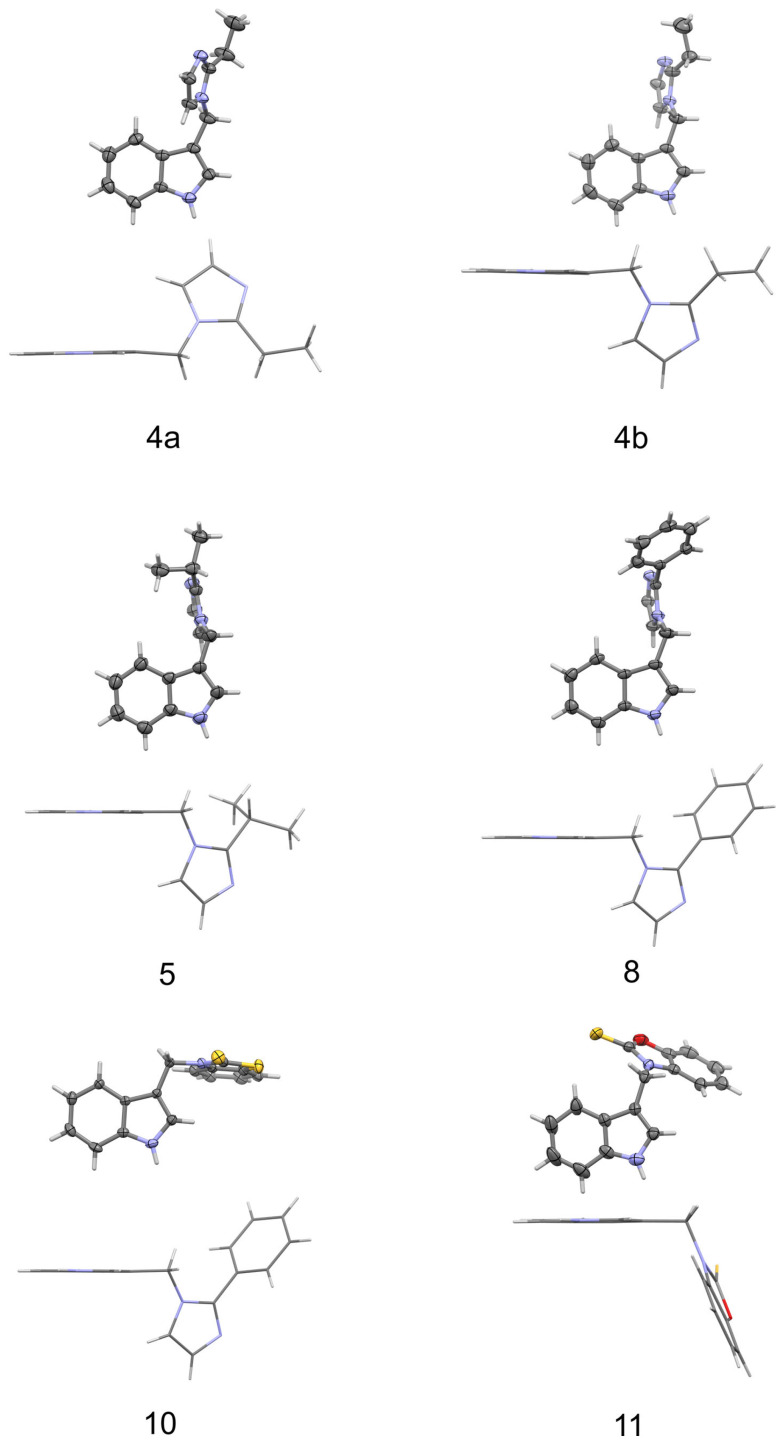
Molecular conformation as present in crystals of compounds **4** (two independent molecules 4a and 4b), **5**, **8**, **10,** and **11** in two representations (i.e., viewed perpendicular (above) and parallel (below) to the indole plane). Thermal ellipsoids are drawn at the 30% probability level.

The structures of the molecules, as seen in the crystals of compounds **4** (two symmetry independent molecules), **5**, **8**, **10**, and **11**, are shown in Figure 3. The main skeleton can be described as consisting of two methylene bridged subunits, each containing aromatic rings which are inclined with respect to each other at angles varying from 66.40(4) to 84.20(3)°. One of the fragments is always a planar indole moiety, while the other is either 2-substituted imidazole (**4**, **5**, **8**) or a planar benzothiazole-2-thione (**10**) or benzoxazole-2-thione (**11**). In molecules **4**, **5,** and **8**, the indole part forms an angle of 62.7(2), 63.6(2), 65.6(1), and 61.4(1)° with a plane containing C-C and C-N bonds to the methylene group (called the methylene plane), while the imidazole fragment is inclined to this plane at only 10.3(1), 13.2(1), 31.3(2) and 28.0(2)°. 

Moreover, a comparison of two independent molecules in crystal of **4** (**4a** and **4b** shown in Figure 3) shows that the two molecules are conformationally enantiomeric. This relationship can also be seen in the values of the torsion angles φ_1_ and φ_2_ measured along the C-C-C-N and C-C-N-C methylene bonds that are listed in Table 1. As one can see from this table, both torsion angles in a molecule always have the same sign, which is the condition for a propeller conformation. Due to the fact that the crystals are predominantly centrosymmetric (with the exception of **10**, which crystallizes in Sohncke space group *P*2_1_2_1_2_1_) both types of propellers are uniformly distributed in the investigated crystal structures. 

**Figure 4:**
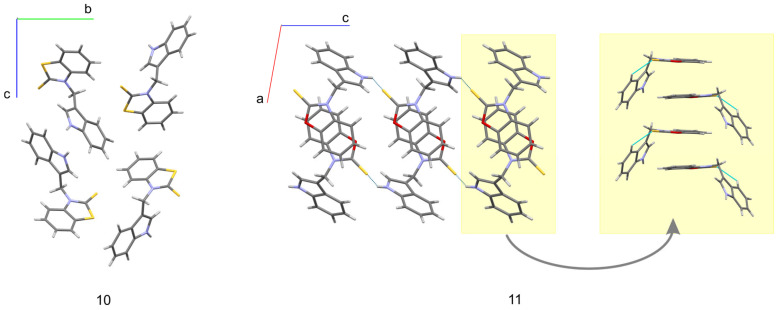
Effect of chalcogen exchange on the solid-state structures of **10** (view along [100]) and **11** (view along [010]).

The molecular conformation markedly changes with the replacement of imidazole fragments by either benzothiazole-2-thione (**10**) or benzoxazole-2-thione (**11**). While in the imidazole derivatives (**4**, **5**, and **8**), the imidazole ring lies almost in the methylene plane, and the indole fragment is significantly out of this plane, in **10,** on the contrary, the indole ring is close to the methylene plane while the benzothiazole-2-thione moiety is out of this plane, the corresponding angles being 13.4(3) and 78.2(1)°. Such conformation is particularly well suited for the formation of an intramolecular hydrogen bond from the methylene C-H donor to the S=C acceptor. The C···S and H···S distances are 3.140(3) and 2.69 Å, and the C-H···S angle amounts to 109°. In **11** both rings are nearly perpendicular to the methylene plane and form angles of 89.7(1) and 80.8(1)° with this plane. This conformation is exceptional for the investigated series and is less favorable for the formation of C-H···S intramolecular hydrogen bond. As follows from the above, the molecules of **10** and **11** differ in conformation not only with respect to the other molecules in the series studied but also between each other (although the chemical difference between them arises solely from the chalcogen exchange). The observed significant alteration of the molecular conformation in the solid state caused by the S/O replacement has far reaching consequences with respect to intermolecular interactions. While in the crystals of **10** the donor N-H group is engaged in intermolecular N-H···π interactions to the benzene part of the indole ring (Table 2), in the crystals of **11** we observe N-H···S=C hydrogen bonds joining the molecules along *c*-direction (Table 2) and columnar stacking interactions between benzoxazole-2-thione fragments, related by the two-fold screw axis along *b*-direction (Figure 4). The distance between the centroids of the two fragments in a stack amounts to 3.658 Å, while the distance between their planes is 3.603 Å (symmetry code –x + 1, y + 1/2, −z + 1/2). The planes are nearly parallel, the interplanar angle being 10° and the displacement of the rings in a stack is only 0.399 Å. 

As expected, indole/imidazole derivatives **4**, **5**, and **8** are mainly engaged in N-H···N hydrogen bonds connecting the molecules into infinite chains (Table 2). 

### 2.3. Antioxidant Properties

#### 2.3.1. Hemolytic Activity

The biocompatibility of the new compounds is one of the main parameters determining their potential biomedical application. One of the main tests to determine the toxicity of compounds is their hemolytic activity. Hence, erythrocytes were used as model cells to test the biocompatibility of compounds **2**–**11** [40].

**Figure 5:**
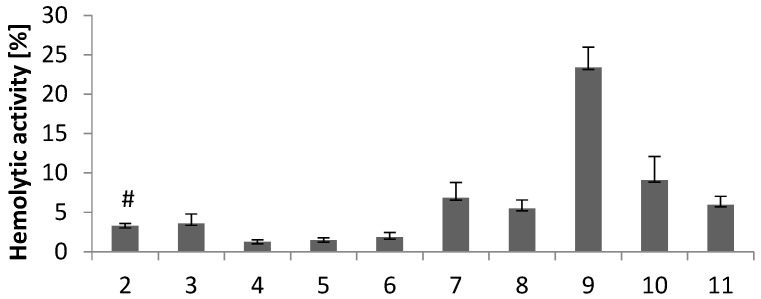
Hemolytic activity of compounds **2**–**11** at a concentration of 0.1 mg/mL. Results (*n* = 7) are presented as the mean value ± standard deviation. # Data published in [18].

The hemolytic activity of all derivatives was evaluated in vitro using human red blood cells (RBC). As shown in Figure 5, the hemolytic activity of the tested compounds (0.1 mg/mL) depends on their chemical structure (compare Figure 5 with Figure 2). The hemolytic activity of indole derivatives with electron-donating substituents at the imidazole ring (**3**–**6**) was below 5%. This means that **3**–**6** are biocompatible compounds without the cell membrane-disrupting activity and are good candidates for further evaluation. The hemolytic activity of compound 3 (3.6%) with a methyl substituent at the imidazole ring is comparable to that of derivative **2** with unsubstituted imidazole (3.3%). 

The electron-withdrawing substituents increase the hemolytic activity of compounds in the order of **9** > **10** > **7** > **11** > **8**. The hemolytic activity of compounds **7**, **8**, **10**, and **11** ranges from 5.5% to 9.1%. Compound 9 with a benzimidazole moiety has the highest hemolytic activity (23%). According to the above results, compounds **7**–**11** are not biocompatibile at the concentration tested. 

#### 2.3.2. Cytoprotective Activity against Oxidative Stress

Oxidative stress is an imbalance between the production of reactive oxygen species (ROS) and the efficiency of the antioxidant system. Overproduction of ROS are associated with cancer, cardiovascular, neurodegenerative, and autoimmune diseases [41]. A high levels of ROS can lead to lipid peroxidation, protein aggregation, and nucleic acid damage. 

Exogenous antioxidants have received much attention in recent years, as they may play an important role in preventing oxidative damage in cells. Indole derivatives are known for their antioxidant properties, including protection of cells from ROS detrimental effects [18,19,20]. The high reactivity of indole antioxidants is probably due to the electron-rich aromatic ring system, which enables them to act as electron donors for the formation of cationic radicals or by the addition of electrophilic radicals at the C-3 position of the indole [16]. 

**Figure 6:**
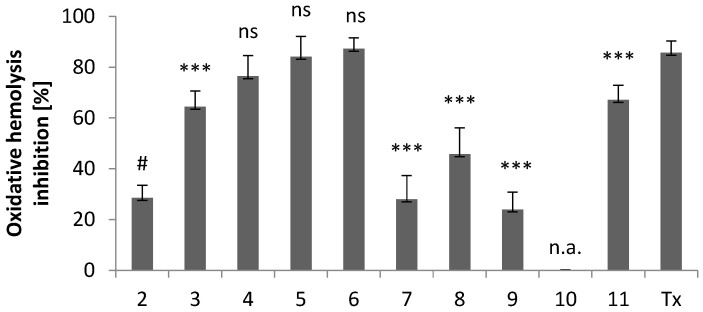
Cytoprotective activity of compounds **2**–**11** and the standard antioxidant Trolox against oxidative hemolysis induced by free radicals generated from AAPH. The results (*n* = 7) are presented as the mean value ± standard deviation (*** *p* < 0.001) in comparison with the standard antioxidant Trolox. Non statistically significant difference (*p* > 0.05) is indicated as ns. Inactive compounds are indicated as n.a. # Data published in [18].

As shown in Figure 6, the cytoprotective activity of new derivatives at a concentration of 0.1 mg/mL strongly depends on the substituent present at the C-3 position of the indole ring. 

In compounds **3**–**8**, the electron-withdrawing or electron-donating properties of the substituent present in the imidazole ring are of great importance. For derivatives with electron-donating groups, the cytoprotective activity increases in the order of substituents: methyl and ethyl (**6**) > isopropyl (**5**) > ethyl (**4**) > methyl (**3**). Compound **7** with electron-withdrawing substituents exhibits cytoprotective activity similar to the unsubstituted imidazole derivative (**2**). A similar cytoprotective activity is observed for compound **9**. A slightly higher value of cytoprotective activity is observed for the derivative with a phenyl ring (**8**).

The antioxidant properties of compounds **2**–**9** are related to electrons delocalization in the imidazole and indole rings. The indole rings act as electron or hydrogen atom donors. When an electron is lost, a stabilized radical cation is formed. It is also possible to transfer a hydrogen atom from the antioxidant molecule to the radical and form a resonance-stabilized indolyl radical. 

The results showed that the most important structural element affecting the cytoprotective activity of the compounds studied is the presence of alkyl substituents in the imidazole ring. The imidazole ring acts as the second electron-rich donor. The alkyl substituents contribute to the delocalization of radicals obtained and provide necessary stability to the system. 

Particularly noteworthy is the comparison of the cytoprotective activity of derivatives **10** and **11**. While compound **10**, with benzothiazole-2-thione moiety, did not protect RBC from oxidative stress-induced hemolysis, derivative **11** with benzoxazole-2-thione scaffold showed significant cytoprotective activity. We are inclined to attribute this major difference in activity to the structural changes that take place upon replacement of -S- by –O- ring atom *(vide supra*). The changes involve both the molecular and supramolecular levels in the crystals, but may also reflect ligand/receptor interactions in the process of molecular recognition.

#### 2.3.3. Chelating Activity

One measure of the compounds’ antioxidant properties is their capacity to chelate iron ions. Chelating compounds prevent the ability of the iron to catalyze the Haber-Wiess or Fenton-type reactions leading to hydroxyl radical formation. Depending on the conditions, biologically inactive ferric ions (Fe^3+^) can be reduced to active ferrous ions (Fe^2+^) and then oxidized back with the generation of ROS, leading to neurodegenerative diseases such as—Parkinson’s disease, Huntington’s disease, Alzheimer’s disease, and schizophrenia [42]. 

**Figure 7:**
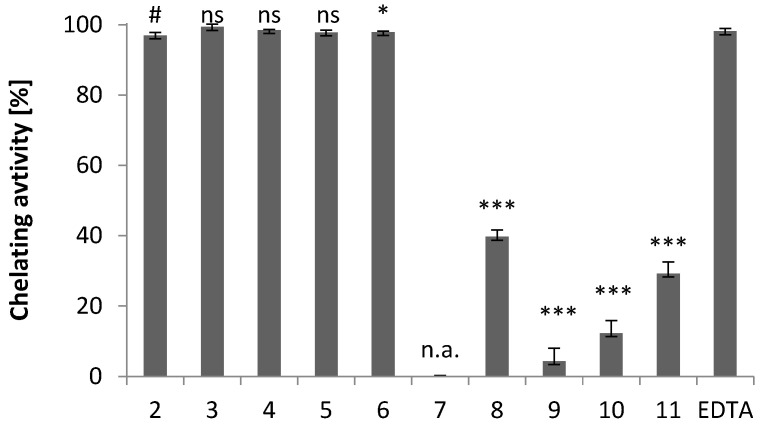
Ferrous ion chelating activity of iron ions of compounds **2**–**11** and the standard chelating agent EDTA. The results (*n* = 3) are presented as the mean value ± standard deviation (* *p* < 0.05, *** *p* < 0.001) in comparison with EDTA. Non statistically significant difference (*p* > 0.05) is indicated as ns. Inactive compounds are indicated as n.a. # Data published in [18].

As shown in Figure 7, the ferrous chelating properties of the compounds studied are depend on their chemical structure (compare Figure 2 with Figure 7). Among the indole-imidazole derivatives (**2**–**9**), compounds **2**–**6** are the most effective as ferrous ion chelators, and their complexing ability is comparable to EDTA, used as a standard chelator. The chelating activity of compounds **3**–**6** is comparable to that of compound **2**. The presence of an electron-donating alkyl substituent in the imidazole ring does not alter the chelating activity of the compounds. 

Compound **7** has no chelating activity, while the chelating activities of compounds **8** and **9** are 40% and 4%, respectively (Figure 7). This reduction in chelating activity can be attributed to the electron-withdrawing substituents, which reduce the electron density on the imidazole nitrogen atom and thus reduce the metal-ligand interaction.

The chelating activity values for compounds **10** and **11** are 12% and 29%, respectively. The lack of intermolecular hydrogen bonds to the thiolate sulfur atom in the crystals of **10** suggests that this atom is not readily available for interactions with the neighboring molecules, and thus also for metal-ligand interactions.

### 2.4. Antibacterial Study

The bacteria selected for this study (*Bacillus subtilis*, *Micrococcus luteus*, *Escherichia coli,* and *Pseudomonas fluorescens*) belong to the model species, due to their widespread occurrence in the natural environment, being a natural component of the human opportunistic microbiome [43,44,45].

Among all tested compounds, compound **2** showed the highest antimicrobial activity, for which the largest growth-inhibition zones were noted, particularly evident in the *M. luteus* (21 mm) and *P. fluorescens* (14.7 mm) cultures (Table 3). Compounds **4** and **8** also showed strong inhibitory effects on the tested bacteria, but the latter did not inhibit the growth of *P. fluorescens*. The weakest antagonists of the tested bacteria turned out to be two compounds—gramine (**1**) and their derivative **10**. Gramine had a potent inhibitory effect only on the *P. fluorescens* strain, while it had the least effect on the growth of the population of other bacterial species. In contrast, derivative **10** only slightly inhibited the growth of *M. luteus* and *E. coli*.

### 2.5. Fungicidal Activity

Four fungal strains (*Coriolus versicolor (Cv), Poria placenta (Pp), Coniophora puteana (Cp),* and Gloeophyllum trabeum (Gt)) were used to study the antifungal activity of gramine (1) and their derivatives (2–11). These selected indoor, wood-destroying fungi contribute significantly to wood degradation [46] and are widely used in standard and custom mycological tests. 

As shown in Table 4, the effective dose, ED100, for all tested compounds (i.e., no mycelial growth), occurs when 0.1% of the alkaloid is present in the agar medium. At a concentration of 0.01%, four derivatives (**7**, **8**, **10**, **11**) show fungistatic activity. Among them, the indole-imidazole compound with chlorine atoms (**7**) demonstrated the strongest fungicidal effects against all tested fungal strains. Its 0.1% solution inhibited the growth of *C. *versicolor** and *G. trabeun* by more than 90% and *C. puteana* and *P. placenta* by more than 70%. Derivative **10** with benzothiazole moiety showed a 71, 59, and 57% inhibition effect against the mycelia elongation of *P. placenta*, *G. trabeun,* and *C. puteana*, respectively. In contrast, benzoxazole derivative (**11**) inhibits only one strain of fungi *P. placenta* (64% inhibition). Compound **8** was the most potent against *G. trabeun* (53% inhibition). The other compounds tested were ineffective at this concentration. 

It is worth noting that of all compounds tested, three (**7**, **10**, **11**) showed fungicidal activity against *C. puteana* and *P. placenta*, which are highly resistant to currently used fungicides.

### 2.6. In Silico Study

The SwissADME website [47] was used to calculate the physicochemical and pharmacokinetic properties of the compounds and their drug-likeness. As shown in Table 5, all compounds investigated meet the criteria of Lipinski’s rule of five. The molecular mass (MW) is less than 500 g/mol, the partition coefficient (logP) is less than 5, and there are no more than 5 hydrogen bond donors (HBD) and 10 hydrogen bond acceptors (HBA) [48]. Lipophilicity is one of the descriptors of xenobiotics, that influences their biological properties, affecting the bioavailability, biodegradation, and toxicity of substances [49]. The alkyl substitution of the imidazole ring in compounds **3**–**6** improves their lipophilicity and facilitate incorporation into the cell membrane, making them excellent cytoprotective agents. LogP values of 2.20–2.84 for derivatives **3**–**6** appear ideal for passing the biological membrane. The partition coefficient calculated for this group of indole derivatives correlates well with their structural features—the longer the alkyl chain, the higher values of the partition coefficient. Of all compounds tested, derivative **10** is the most lipophilic (logP = 4.13). All derivatives analyzed have one hydrogen bond donor (HBD), and most have one hydrogen bond acceptor (HBA). 

The results showed that none of the synthesized compounds violated Veber’s rule, suggesting good oral bioavailability. It depends on the number of rotatable bonds (RTB) and the topological polar surface area (TPSA) [50]. The number of rotatable bonds influences, apart from bioavailability, the binding potency of compounds should be less than 10. As shown in Table 5, all analyzed derivatives have 2 or 3 RTB. 

TPSA is a measure of the ability of a drug to pass cell membrane. A TPSA value of less than 140Å2 is characteristic of the passive transport of the molecule across cell membrane. Most of the indole derivatives obtained have TPSA values less than 34.

The pharmacokinetic properties of compounds involve various factors, among which GI (Gastrointestinal) absorption and BBB (Blood-Brain Barrier) permeation are important. The more a dose reaches the bloodstream after oral administration (mainly from the gastrointestinal tract), the more it can cross the BBB by passive diffusion [51]. All compounds investigated show high gastrointestinal absorption and can cross the BBB with the exception of 10. Although a derivative with benzothiazole-2-thione can be absorbed from the gastrointestinal tract, it cannot cross the BBB, likely due to its poor water solubility and high lipophilicity.

One of the main challenges of obtaining new potential drugs is the synthesis of a water-soluble product. A compound with higher water solubility is more bioavailable and requires a lower dose to reach a therapeutic plasma concentrations after oral administration [52]. Depending on the calculation method, the water solubility of compounds 2–9 is good or moderate (Table 6). As the most lipophilic, derivatives 10 and 11 are also the least soluble in water, showing moderate or poor solubility. 

The results obtained suggest that the indole-imidazole derivatives **3**–**6** have good ADME parameters and can be considered as good candidates for the development of novel antioxidants. 

## 3. Materials and Methods

### 3.1. Instrumentation and Chemicals

The melting points (mp) were obtained with a Büchi SMP-20 apparatus. ^1^H NMR and ^13^C NMR spectra were recorded on a Varian 300/400 spectrometer with DMSO-*d_6_* as the solvent and TMS as the internal standard. Chemical shifts are reported in δ (parts per million) values. EI mass spectra were measured on Bruker 320MS/450GC mass spectrometer. FT-IR spectra were recorded on Nicolet iS 5 (KBr pellets). TLC analysis was used using Sigma-Aldrich silica gel 60 plates with a fluorescent indicator (254 nm) and visualized with UV. All chemicals or reagents used for syntheses were commercially available. In all reactions, anhydrous solvents were used. 

### 3.2. Synthesis of Gramine Derivatives

Synthesis of compound **2** was already described in our previous paper [18].

***A typical procedure for the synthesis of compounds 3–11***.

A solution of gramine (1 mmol) and the appropriate nucleophilic compound (1 mmol) in 8–10 mL of toluene was heated under reflux for 2–10 h. After completion of the reaction, as indicated by TLC, the precipitate was filtered and crystallized from toluene.

3-((2-methyl-1H-imidazol-1-yl)methyl)-1H-indole (**3**)

Light yellow solid (180 mg, 85%); m.p. 173–174 °C; ^1^H NMR (400 MHz, DMSO-d_6_): δ 11.14 (s, 1H), 7.50 (d, J = 7.9 Hz, 1H), 7.39–7.37 (m, 2H), 7.12–7.07 (m, 2H), 6.99 (ddd, J = 8.0, 7.0, 1.0 Hz, 1H), 6.68 (d, J = 1.3 Hz, 1H), 5.21 (s, 2H), 2.33 (s, 3H); ^13^C NMR (101 MHz, DMSO-d_6_): δ 143.56, 136.32, 126.05, 125.85, 126.63, 121.41, 119.98, 118.92, 118.30, 111.67, 110.40, 40.99, 13.01; IR (KBr): 3550–3460, 3139–2776, 1524, 1455, 1427, 1360, 750; EI-MS (*m/z*, % int.): 211 (M^+^, 9), 130 (100). Analysis calculated for C_13_H_13_N_3_ (MW = 211.26): C 73.91, H 6.20, N 19.89; found: C 73.62, H 6.25, N 19.87%.

3-((2-ethyl-1H-imidazol-1-yl)methyl)-1H-indole (**4**)

White solid (225 mg, 89%); m.p. 183–186 °C; ^1^H NMR (400 MHz, DMSO-*d_6_*) δ 11.12 (s, 1H), 7.47 (d, *J* = 7.9 Hz, 1H), 7.37–7.41 (m, 2H), 7.14–7.08 (m, 2H), 6.98 (ddd, *J* = 8.0, 7.0, 1.1 Hz, 1H), 6.71 (d, *J* = 1.3 Hz, 1H), 5.22 (s, 2H), 2.69 (q, *J* = 7.5 Hz, 2H), 1.16 (t, *J* = 7.5 Hz, 3H); ^13^C NMR (101 MHz, DMSO-*d_6_*) δ 148.10, 136.31, 126.03, 125.81, 124.57, 121.38, 119.88, 118.89, 118.27, 111.65, 110.46, 40.74, 19.59, 12.01; IR (KBr): 3432, 3131–2612, 1455, 1422, 1379, 781; EI-MS (*m/z*, % int.): 225 (M^+^, 35), 130 (100). Analysis calculated for C_14_H_15_N_3_ (MW = 225.29): C 74.64, H 6.71, N 18.65; found: C 74.26, H 6.75, N 18.53%.

3-((2-isopropyl-1H-imidazol-1-yl)methyl)-1H-indole (**5**)

Light yellow solid (239 mg, 88%); m.p. 183–185 °C; ^1^H NMR (400 MHz, DMSO-d_6_) δ 11.12 (s, 1H), 7.45 (d, J = 8.0 Hz, 1H), 7.38 (dt, J = 8.2, 0.8 Hz, 1H), 7.32 (d, J = 2.5 Hz, 1H), 7.09 (ddd, J = 8.2, 7.1, 1.1 Hz, 1H), 7.02–6.98 (m, 2H), 6.71 (d, J = 1.2 Hz, 1H), 5.25 (s, 2H), 3.23 (hept, J = 6.7, 1H), 1.15 (d, J = 6.8, 6H); ^13^C NMR (101 MHz, DMSO-d_6_) δ 151.88, 136.30, 126.02, 125.77, 124.48, 121.38, 119.48, 118.88, 118.27, 111.65, 110.67, 40.68, 25.14, 21.98 (2x); IR (KBr): 3415, 3133–2872, 2613, 1875, 1488, 1432, 1379, 741; EI-MS (*m/z*, % int.): 239 (M^+^, 30), 130 (100). Analysis calculated for C_15_H_17_N_3_ (MW = 239.32): C 75.28, H 7.16, N 17.56; found: C 75.17, H 7.23, N 17.64%.

3-((2-ethyl-4-methyl-1H-imidazol-1-yl)methyl)-1H-indole (**6**)

Light yellow solid (239 mg, 69%); m.p. 186–189 °C; ^1^H NMR (400 MHz, DMSO-d_6_) δ 11.08 (s, 1H), 7.44 (d, J = 7.9 Hz, 1H), 7.33 (d, J = 2.4 Hz, 1H), 7.06 (ddd, 1H), 6.99–6.93 (m, 2H), 6.69 (d, J = 0.9 Hz, 1H), 5.09 (s, 2H), 2.66–2.60 (m, 2H), 1.95 (s, 3H), 1.10 (t, J = 7.5 Hz, 3H) ^13^C NMR (101 MHz, DMSO-d_6_) δ 147.30, 136.30, 133.93, 126.08, 124.54, 121.36, 118.89, 118.28, 115.91, 111.67, 110.65, 40.37, 19.53, 13.60, 12.15, 9.61; IR (KBr): 3422, 3136–2761, 1457, 1420, 1379, 777; EI-MS (*m/z*, % int.): 239 (M^+^, 75), 130 (100). Analysis calculated for C_15_H_17_N_3_ (MW = 239.32): C 75.28, H 7.16, N 17.56; found: C 75.19, H 7.18, N 17.62%.

3-((4,5-dichloro-1H-imidazol-1-yl)methyl)-1H-indole (**7**)

Dark pink oil (133 mg, 50%); ^1^H NMR (400 MHz, DMSO-d_6_) δ 11.22 (s, 1H), 7.97 (s, 1H), 7.62–7.57 (m, 1H), 7.48 (d, J = 2.6 Hz, 1H), 7.42–7.38 (m, 1H), 7.15–7.09 (m, 1H), 7.03 (ddd, J = 10.2, 5.6, 2.1 Hz, 1H), 5.34 (s, 2H).; ^13^C NMR (101 MHz, DMSO-d_6_) δ 136.20, 135.93, 128.88, 128.19, 125.71, 125.30, 121.58, 119.18, 117.99, 111.81, 108.34, 41.46. IR (KBr): 3411, 3057, 2927, 1456, 1252, 743 cm^−1^; EI-MS (*m/z*, % int.): 265 (M^+^, 3), 130 (100). Analysis calculated for C_12_H_9_N_3_Cl_3_ (MW = 266.13): C 54.16, H 3.41, N 15.79; found: C 54.21, H 3.50, N 15.83%.

3-((2-phenyl-2,5-dihydro-1H-imidazol-1-yl)methyl)-1H-indole (**8**)

Yellow solid (180 mg, 56%); m.p. 110–120 °C (with decomposition); ^1^H NMR (400 MHz, DMSO-d_6_) δ 11.09 (s, 1H), 7.72–7.64 (m, 2H), 7,54–7.39 (m, 3H), 7.35 (d, J = 8.1 Hz, 1H), 7.29–7.15 (m, 2H), 7.14 (d, J = 2.5 Hz, 1H), 7.13–7.03 (m, 1H), 6.99–6.86 (m, 2H), 5.42 (s, 2H); ^13^C NMR (101 MHz, DMSO-d_6_) δ 146.44, 136.19, 131.18, 128.59 (2×), 128.51 (2×), 128.39, 127.70, 125.81, 124.37, 121.85, 121.44, 118.95, 117.96, 111.68, 110.53, 42.34; IR (KBr): 3420, 3151–3059. 2982–2869, 1458, 1376, 1234 cm^−1^; EI-MS (*m/z*, % int.): 144 (M^+^, 17), 130 (100). Analysis calculated for C_19_H_15_N_3_ (MW = 273.33): C 78.52, H 6.22, N 15.26; found: C 78.48, H 6.19, N 15.31%.

1-((1H-indol-3-yl)methyl)-1H-benzo[d]imidazole (**9**)

White solid (235 mg, 95%); m.p. 214–216 °C; ^1^H NMR (400 MHz, DMSO-d_6_): δ 11.14 (s, 1H), 8.45 (s, 1H), 7.70 (dt, J = 8.0, 1.0 Hz, 1H), 7.65–7.54 (m, 3H), 7.36 (dt, J = 8.1, 0.9 Hz, 1H), 7.25–7.10 (m, 2H), 7.07 (ddd, J = 8.1, 7.0, 1.2 Hz, 1H), 6.97 (ddd, J = 8.0, 7.1, 1.0 Hz, 1H), 5.61 (s, 2H); ^13^C NMR (101 MHz, DMSO-d_6_) δ 144.07, 143.62, 136.32, 133.71, 126.08, 125.33, 122.03, 121.40, 121.30, 119.34, 118.96, 118.25, 111.70, 110.89, 109.65, 39.89; IR (KBr): 3400–3000, 2927–2868, 1934, 1456, 1351, 1265, 740 cm^−1^; EI-MS (*m/z*, % int.): 247 (M^+^, 4), 130 (100). Analysis calculated for C_16_H_13_N_3_ (MW = 247.29): C 77.71, H 5.30, N 16.99; found: C 77.68, H 5.27, N 17.07%.

3-((1H-indol-3-yl)methyl)benzo[d]thiazole-2(3H)-thione (**10**)

Light yellow Solid (296 mg, 75%); m.p. 187–190 °C; ^1^H NMR (400 MHz, DMSO-d_6_): δ 11.17 (s, 1H), 7.84 (dd, J = 8.0, 1.1 Hz, 1H), 7.74 (d, J = 2.5 Hz, 1H), 7.68 (m, 2H), 7.59 (d, J = 2.5 Hz, 1H), 7.43–7.29 (m, 1H), 7.27–7.24 (m, 2H), 7.08 (m, 1H), 5.90 (s, 2H); ^13^C NMR (101 MHz, DMSO-d_6_) δ 188.32, 141.20, 136.20, 127.02, 126.51, 125.94, 125.86, 124.94, 121.81, 121.58, 119.11 (2X), 114.21, 111.78, 108.22, 42.22; IR (KBr): 3563, 3397, 3075, 3054, 1460, 1368, 747 cm^−1^; EI-MS (*m/z*, % int.): 296 (M^+^, 68), 130 (100). Analysis calculated for C_16_H_12_N_2_S_2_ (MW = 296.41): C 64.84, H 4.08, N 9.45, S 21.63; found: C 64.91, H 4.12, N 9.51, S 21.68%.

3-((1H-indol-3-yl)methyl)benzo[d]oxazole-2(3H)-thione (**11**)

Brown solid (280 mg, 75%); m.p. 157–159 °C; ^1^H NMR (400 MHz, DMSO-d_6_): δ 11.23 (s, 1H), 7.84 (d, J = 8.1 Hz, 1H), 7.73 (d, J = 2.5 Hz, 1H), 7.58–7.50 (m, 2H), 7.43–7.38 (m, 3H), 7.15–7.09 (m, 2H), 5.64 (s, 2H); ^13^C NMR (101 MHz, DMSO-d_6_) δ 179.04, 146.40, 136.23, 131.13, 126.55, 125.93, 124.93, 124.33, 121.57, 119.11, 118.94, 111.74, 111.37, 110.11, 107.50, 41.79; IR (KBr): 3407, 3111, 3058, 2942, 1490, 1423, 775 cm^−1^; EI-MS (*m/z*, % int.): 280 (M^+^, 63), 130 (100). Analysis calculated for C_16_H_12_N_2_SO (MW = 280.34): C 68.55, H 4.31, N 9.99, S 11.14; found: C 68.51, H 4.27, N 10.07, S 11.16%. 

### 3.3. X-Ray Analysis

Single–crystal X-ray diffraction measurements were carried out with the monochromated CuK_α_ radiation on a SuperNova diffractometer or MoK_α_ radiation on an Xcalibur diffractometer. The data were collected and processed using the CrysAlis^Pro^ software [53]. The crystal structures were solved by direct methods with SHELXT [54] and refined by full-matrix least-squares calculations on F^2^ with SHELXL [55]. All non-H atoms were refined with anisotropic displacement parameters. Hydrogen atoms were placed at calculated positions based on the environment and perceived hybridization of the C atoms to which they are bonded (methyl C–H = 0.96 Å, methylene C–H = 0.97 Å, aromatic C-H = 0.93 Å and N-H = 0.86 Å) and refined as ‘riding’ on their carriers. During the refinement, isotropic displacement parameters for H-atoms were assigned 20% higher than the isotropic equivalent for the atom to which the H-atom was bonded. In crystals of **4** there were signs of disorder of the terminal ethyl groups. We have modelled the disorder for one of the two ethyl substituents by taking into account two alternative positions for the methylene group. During the refinement the occupancy ratios for the major and minor component were negatively coupled so the total occupancy remained equal to one. The component occupancy factors amount to 0.55 and 0.45, respectively. The final model is not fully satisfactory, namely the atomic displacement parameters for atoms constituting ethyl substituents are relatively high and the C-C bonds to these atoms appear to be shorter than expected. MERCURY [56] computer graphics programs were used to prepare drawings. The crystal data together with experimental and refinement details are collected in Table 7. CCDC 2223465-2223469 contains the supplementary crystallographic data for this paper (for structures **4**, **5**, **8**, **10** and **11**, respectively). These data can be obtained free of charge via http://www.ccdc.cam.ac.uk/conts/retrieving.html (or from the CCDC, 12 Union Road, Cambridge CB2 1EZ, UK; Fax: +44-1223-336033; E-mail: deposit@ccdc.cam.ac.uk.

### 3.4. Antioxidant Study 

#### 3.4.1. Ferrous Ions (Fe^2+^) Chelating Activity

Ferrous ions (Fe^2+^) chelating activity was evaluated by inhibition of the formation of Fe^2+^-ferrozine complex after incubation of the compounds tested with Fe^2+^. The Fe^2+^-chelating ability of compounds tested was determined by the absorbance of the ferrous ion-ferrozine complex at 562 nm. In brief, 0.1 mg/mL concentration of the compounds tested in 0.2 mL ethyl alcohol were added to a solution of 0.6 mM FeCl_2_ (0.05 mL). EDTA (ethylenediaminetetraacetic acid) was used as the standard EDTA chelating agent. The reaction was started by the addition of 5 mM ferrozine (0.05 mL) in ethyl alcohol and shaken vigorously immediately. The samples were stored for 10 min at room temperature (~22 °C). Following incubation, the absorbance (Abs) of the solutions was measured at 562 nm in a spectrophotometer. The percentage of inhibition of ferrozine—Fe^2+^ complex formation was calculated using the equation:

Fe^2+^ chelating effect (%) = [1 − (Abs_1_/Abs_0_)] × 100%

where Abs_0_ is the absorbance of the sample without the tested compound and Abs_1_ is the absorbance in the presence of the compound tested. Each sample was made in triplicate, and three independent experiments were performed.

#### 3.4.2. Human Erythrocyte Preparation

All methods were carried out following relevant guidelines and regulations, and the Bioethics Committee approved all experimental protocols for Scientific Research at the Medical University of Poznań (agreement no. ZP/907/1002/18). Human RBC concentrates were purchased from Blood Bank in Poznań without any contact with blood donors. 

The erythrocytes were washed three times (3000 rpm, 10 min, +4 °C) in 7.4 pH phosphate buffered saline (PBS: 137 mM NaCl, 2.7 mM KCl, 10 mM Na_2_HPO_4_, 1.76 mM KH_2_PO_4_) supplemented with 10 mM glucose. After washing, RBC were suspended in the PBS buffer at 1.65 × 10^9^ cells/mL, stored at +4 °C, and used within 5 h.

#### 3.4.3. Hemolysis Assay under the Compounds Tested

The cytotoxic activity of the compounds tested was determined by a standard hemolytic assay according to Mrówczyńska and Hägerstrand [57]. Briefly, RBC (1.65 × 10^8^ cells/mL, ~1.5% hematocrit) were incubated in PBS buffer (7.4 pH) supplemented with 10 mM glucose and containing compounds tested (0.1 mg/mL) for 60 min at 37 °C in a shaking water bath. Samples with RBC incubated in PBS without compounds tested were taken as the control. Each sample was repeated three times, and the experiments were repeated 3 times with RBC from different donors. After incubation, the RBC suspensions were centrifuged (3000 rpm, 10 min), and the degree of hemolysis was estimated by measuring the absorbance (Ab) of the supernatant at 540 nm. The results were expressed as a percentage (%) of hemolysis which was determined using the following equation:

Hemolysis (%) = (sample Ab/positive control Ab) × 100%

were positive control is Ab of supernatant of RBC in ice-cold H_2_O.

#### 3.4.4. Inhibition of the Free-Radical-Induced Hemolysis

RBC (1.65 × 10^8^ cells/mL, ~1.5% hematocrit) were incubated in PBS buffer (pH 7.4) supplemented with 10 mM glucose and containing compounds tested in the sublytic concentration (0.1 mg/mL) for 20 min at 37 °C in a shaking water bath. After pre-incubation, 2,2’-azobis(2-methylpropionamidine) dihydrochloride (AAPH) was added at the final concentration of 60 mM. Samples were incubated for the next 4 h at 37 °C in a shaking water bath. Erythrocytes incubated in PBS only and in the presence of AAPH, were taken as the negative and positive controls, respectively. After incubation, the erythrocyte suspensions were centrifuged (4000 rpm, 5 min, +4 °C), and the degree of hemolysis was determined by measuring the absorbance (Ab) of the supernatant at 540 nm in a spectrophotometer. The percentage of inhibition was calculated using the following equation:

Inhibition of hemolysis (%) = 100 − [(Ab_sample_ − Ab_PBS_/Ab_AAPH_ − Ab_PBS_) × 100%]

where Ab_sample_ is the absorbance value of supernatant obtained from samples incubated with compounds tested in the presence of AAPH, Ab_PBS_ is the absorbance of supernatant obtained from PBS control (samples without compounds tested) and Ab_AAPH_ is the absorbance of supernatant obtained from AAPH controls (without compounds tested). Each sample was made in triplicate, and the results are presented as a mean value ± SD (*n* = 9) value of three independent experiments with RBC from different donors. 

#### 3.4.5. Statistical Analysis

For antioxidant and cytoprotective properties, data were plotted as the mean value ± standard deviation (SD) of the results of three independent experiments, with every sample in triplicate (*n* = 9). A paired t-Student test was used to compare the derivatives activity with the activity of the standard antioxidants Trolox or EDTA, respectively. Statistical significance was defined as *p* < 0.05. Inactive compounds were indicated as na. Non statistically significant difference was indicated as n.s.

### 3.5. Antibacterial Activity Measurements

The antimicrobial properties of compounds were determined against selected bacteria: *Micrococcus luteus*, *Bacillus subtilis*, *Escherichia coli* and *Pseudomonas fluorescens* which came from the collection of Pure Cultures of the Facility of Microbiology of the Department of Soil Science and Microbiology of the Poznan University of Life Sciences. 

The well-diffusion method was used to evaluate the antimicrobial properties of compounds. 6 mL each of liquidized broth medium was poured into sterile Petri dishes and allowed to solidify. After which, two sterile glass rings with a diameter of 0.5 cm were placed on the surface of each plate. Then 20 mL each of liquid broth medium containing suspensions of the tested bacterial strains at a density of 10^7^ cells/cm3, obtained from 48-h cultures on broth slants, was introduced. After the medium solidified, the glass rings were removed with a pencil, obtaining two wells on each plate. 0.1 mL of compound dissolved in pure dimethyl sulphoxide was introduced into one well, and 0.1 mL of pure dimethyl sulphoxide was introduced into the other well, which was as a control. Each compound was tested in four replicates. The plates were incubated for 48 h in a thermostat at 27 °C for *M. luteus*, *B. subtilis* and *P. fluorescens* cultures, and the *E. coli* culture at 37 °C. At the end of the incubation, the diameters of growth inhibition of the tested strains were measured using calipers.

### 3.6. Antifungal Activity Measurements

Fungal strains: *Coriolus versicolor* (Cv), *Poria placenta* (Pp), *Coniophora puteana* (Cp) and *Gloeophyllum trabeum* (Gt) were used as a test organism for the experimental determination of the antifungal activity of **1**–**11**. The fungus were obtained from the collection of the Department of Wood Chemical Technology, Faculty of Forestry and Wood Technology, Poznań, University of Life Sciences, Poland. The fungal growth rates were measured in 90 mm-diameter plastic dishes using the agar-plate method, described by Ważny and Thorton [58]. Five concentrations of gramine and its derivatives in the range of 0.1% to 0.00001% were studied. The compound was applied on the agar (2% agar and 5% malt-extract) prepared on the Petri dish and previously autoclaved. The combination of the compound and fungus was repeated three times. Given sample was centrally inoculated with a 5 mm diameter disc taken from the submargin of 5-day-old malt agar plates. The plates were incubated in darkness at 21 ± 2 °C and 70 ± 5% relative air humidity. The growth duration was determined by the total coverage of the reference plate. The radius of the area covered by the fungus compared with the reference agar plate was employed to calculate the effective doses, ED50, of preservative concentrations retarding the fungal growth rate by 50%.

### 3.7. In Silico Study

The physicochemical calculations were conducted using the SwissADME website: www.swissadme.ch (accessed on 3 October 2022).

## 4. Conclusions

Based on gramine, a compound of natural origin, we have developed an efficient and easy method to obtained indolylmethane derivatives, which are of significant interest due to their biological activities, especially antioxidant properties. Analysis of the structure-activity relationship showed that the newly synthesized compounds having indole fragment joined by the methylene group with alkyl-substituted imidazole moieties exhibit excellent cytoprotective activity and are effective chelators of ferrous ion.

Of particular interest are the results obtained for **10** and **11,** the chemically closely related yet structurally significantly different compounds. The structural disparity of **10** and **11,** observed at both molecular and supramolecular levels, is reflected in their cytoprotective activity, which is absent in **10** and relatively high in **11**. The enhancement of the cytoprotective activity may have its origin in the ability of a molecule to adopt a conformation that maximizes the number and assortment of intermolecular interactions and therefore is particularly well suited for a wide variety of supramolecular interactions, such as ligand/receptor interactions. 

## Figures and Tables

**Table 1:** Torsion angles (◦) describing rotation around the methylene C-C and C-N bonds in molecules present in crystals.

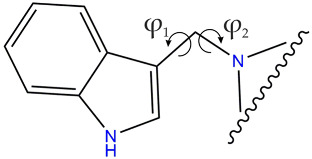		
	**φ_1_**	**φ_2_**
**4a**	66.4 (3)	10.4 (3)
**4b**	−66.3 (3)	−15.1 (3)
**5**	−65.39 (17)	−34.86 (18)
**8**	−62.47 (19)	−31.3 (2)
**10**	14.8 (4)	76.1 (3)
**11**	−88.96 (16)	−80.65 (16)

**Table 2:** Hydrogen bond parameters.

*D*–H···*A*	*D*–H (Å)	H···*A* (Å)	*D*···*A* (Å)	*D*–H···*A* (°)
**4**				
N1–H1···N3^i^	0.86	2.07	2.905 (2)	163
N21–H21···N23^ii^	0.86	2.10	2.915 (2)	157
**5**				
N1–H1···N3^iii^	0.86	2.13	2.953 (1)	159
**8**				
N1–H1···N3^iv^	0.86	2.10	2.957 (1)	173
**10**				
N1–H1···C_g_(C7-C8)^v^	0.86	2.41	3.227	158
**11**				
N1–H1···S1^vi^	0.86	2.78	3.485 (2)	140

Symmetry code(s): (i) *x*−1, *y*, *z*; (ii) *x* + 1, *y*, *z*; (iii) *x*, −*y* + 3/2, *z*−1/2; (iv) *x* + 1/2, −*y* + 3/2, *z* + 1/2; (v) *x−*1/2, *y* + 1/2, −*z +* 1; (vi) *x*, −*y* + 1/2, *z*−1/2; C_g_(C7-C8) designs centre of gravity of the C7-C8 bond.

**Table 3:** Antibacterial activities of compounds **1**–**11**.

Compound	Zone of Growth Inhibition [mm]			
	*Micrococus luteus*	*Bacillus subtilis*	*Escherichia coli*	*Pseudomonas fluorescens*
**1**	0	0	0	13
**2**	21	3.3	4	14.7
**3**	3	1	1	16
**4**	8.8	4.3	3.8	3
**5**	3	1.8	3	0
**6**	6	2.4	3.5	2.3
**7**	5	8.7	4.3	0
**8**	8.6	3.7	5	0
**9**	4.3	2.7	3	0
**10**	2.6	0	1	0
**11**	4	0	2.7	0

**Table 4:** Antifungal activity of compounds **1**–**11**.

Tested Fungi	Concentration	Compound										
	[%]	1	2	3	4	5	6	7	8	9	10	11
*Conipora puteana* (Cp)	0.1	100	100	100	100	100	100	100	100	100	100	100
	0.01	51	41	48	37	26	49	74	48	40	57	44
	0.001	40	0	0	15	14	37	46	0	0	40	43
	0.0001	41	0	0	13	13	40	0	0	0	0	0
*Gleophyllum trabeum* (Gt)	0.1	100	100	100	100	100	100	100	100	100	100	100
	0.01	17	40	44	30	17	27	91	53	48	59	47
	0.001	12	20	19	11	16	9	51	18	28	38	39
	0.0001	12	20	15	8	12	13	28	13	17	0	17
*Poria placenta* (Pp)	0.1	100	100	100	100	100	100	100	100	100	100	100
	0.01	40	47	48	32	21	41	78	45	37	71	64
	0.001	25	20	0	18	6	0	35	17	23	54	56
	0.0001	14	0	0	16	1	4	0	0	0	34	0
*Coriolus versicolor* (Cv)	0.1	100	100	100	100	100	100	100	100	100	100	100
	0.01	35	33	35	43	43	44	96	41	44	49	26
	0.001	22	19	1	13	12	19	23	13	6	20	2
	0.0001	18	0	0	6	13	19	0	7	0	0	0

**Table 5:** Physicochemical, pharmacokinetic and drug-likeness properties of indole derivatives **1**–**11**.

Compound	MW [g/mol]	logP	HBD	HBA	RTB	TPSA [Å^2^]	GI Absorption	BBB Permeation
**1**	174.24	1.94	1	1	2	19.03	high	yes
**2**	197.24	1.82	1	2	2	33.61	high	yes
**3**	211.26	2.21	1	1	2	33.61	high	yes
**4**	225.29	2.49	1	1	3	33.61	high	yes
**5**	239.32	2.82	1	1	3	33.61	high	yes
**6**	239.32	2.84	1	1	3	33.61	high	yes
**7**	266.13	3.07	1	1	2	33.61	high	yes
**8**	273.33	3.26	1	1	3	33.61	high	yes
**9**	247.29	2.92	1	1	2	33.61	high	yes
**10**	296.41	4.13	1	0	2	81.05	high	no
**11**	280.34	3.57	1	1	2	65.95	high	yes

**Table 6:** Water solubility od derivatives **2**–**11** calculated in SwissADME. LogS in the table is the average value of logS calculated using three different methods. * Solubility class—logS scale: Insoluble < −10, Poorly < −6, Moderately < −4, Soluble < −2, Very < 0.

Compound	logS	Solubility [mg/mL]	Solubility Class *
**1**	−2.94	1.18∙10^−2^–2.22	Soluble/very soluble
**2**	−3.36	5.17∙10^−3^–7.20∙10^−1^	Soluble/moderately soluble
**3**	−3.74	2.17∙10^−3^–2.68∙10^−1^	Soluble/moderately soluble
**4**	−3.95	2.15∙10^−3^–1.30∙10^−1^	Soluble/moderately soluble
**5**	−4.10	9.44∙10^−4^–1.10∙10^−1^	Soluble/moderately soluble
**6**	−4.59	9.42∙10^−4^–2.34∙10^−2^	Moderately soluble
**7**	−4.29	3.06∙10^−4^–1.70∙10^−1^	Soluble/moderately soluble
**8**	−4.46	2.62∙10^−4^–7.20∙10^−2^	Soluble/moderately soluble
**9**	−5.44	2.62∙10^−4^–4.89∙10^−3^	Moderately/poorly soluble
**10**	−4.89	2.78∙10^−4^–1.27∙10^−2^	Moderately/poorly soluble
**11**	−2.94	1.18∙10^−2^–2.22	Soluble/very soluble

**Table 7:** Crystal data and structure refinement parameters for selected gramine derivatives.

	4	5	8	10	11
Chemical formula	C_14_H_15_N_3_	C_15_H_17_N_3_	C_18_H_15_N_3_	C_16_H_12_N_2_S_2_	C_16_H_12_N_2_OS
*M*_r_	225.29	239.31	273.33	296.40	280.34
Crystal system, space group	Monoclinic, *P*2_1_/*c*	Orthorhombic, *Pbca*	Monoclinic, *P*2_1_/*n*	Orthorhombic, *P*2_1_2_1_2_1_	Monoclinic, *P*2_1_/*c*
*a*, *b*, *c* (Å)	9.5627(2), 17.1298(3), 15.3143(4)	10.4215(1), 13.5948(2), 18.8911(2)	14.0170(1), 7.0099(1), 15.0277(1)	5.4548(2), 13.9861(3), 18.2075(4)	13.9368(1), 7.2337(1), 13.7132(1)
α, β, γ (°)	90, 105.097(2), 90	90, 90, 90	90, 100.994(1), 90	90, 90, 90	90, 100.242(1), 90
*V* (Å^3^)	2422.01(9)	2676.46(5)	1449.49(3)	1389.08(7)	1360.46(2)
*Z*	8	8	4	4	4
*D_x_* (Mg m^−3^)	1.236	1.188	1.253	1.417	1.369
Radiation type	Cu *K*α	Cu *K*α	Cu *K*α	Mo *K*α	Cu *K*α
μ (mm^−1^)	0.59	0.56	0.59	0.37	2.08
Crystal size (mm)	0.60 × 0.08 × 0.07	0.60 × 0.25 × 0.15	0.30 × 0.20 × 0.05	0.60 × 0.10 × 0.08	0.60 × 0.20 × 0.08
Data collection					
*T*_min_, *T*_max_	0.727, 1.000	0.553, 1.000	0.792, 1.000	0.926, 1.000	0.424, 1.000
No. of measured, independent and observed [*I* > 2 s(*I*)] reflections	39,931, 5059, 4028	25,903, 2798, 2439	24,636, 3027, 2682	36,290, 3350, 2871	33,085, 2857, 2628
*R*_int_	0.054	0.033	0.026	0.039	0.044
(sin θ/λ)_max_ (Å^−1^)	0.632	0.631	0.630	0.671	0.630
Refinement					
*R*[*F*^2^ > 2 s(*F*^2^)], *wR*(*F*^2^), *S*	0.071, 0.239, 1.08	0.042, 0.128, 1.06	0.038, 0.108, 1.06	0.044, 0.083, 1.15	0.042, 0.131, 1.10
No. of reflections	5059	2798	3027	3350	2857
No. of parameters	318	165	191	181	182
No. of restraints	26	0	0	0	12
Δρ_max_, Δρ_min_ (e Å^−3^)	0.51, −0.51	0.11, −0.18	0.13, −0.16	0.19, −0.18	0.20, −0.32
Absolute structure parameter	–	–	–	0.00 (2)	–

## Data Availability

Not applicable.

## Supplementary Materials

The following supporting information can be downloaded at: https://www.mdpi.com/article/10.3390/molecules28020708/s1, Figure S1: (a) ^13^C NMR spectrum (101 MHz, DMSO-*d_6_*): compound **3**, (b) ^1^H NMR spectrum (400 MHz, DMSO-*d_6_*): compound **3;** Figure S2: EI-MS spectrum: compound **3;** Figure S3: IR spectrum: compound **3**; Figure S4: (a) ^13^C NMR spectrum (101 MHz, DMSO-*d_6_*): compound **4**, (b) ^1^H NMR spectrum (400 MHz, DMSO-*d_6_*): compound **4**; Figure S5: EI-MS spectrum: compound **4;** Figure S6: IR spectrum: compound **4;** Figure S7: (a) ^13^H NMR spectrum (101 MHz, DMSO-*d_6_*): compound **5**, (b) ^1^H NMR spectrum (400 MHz, DMSO-*d_6_*): compound **5**; Figure S8: EI-MS spectrum: compound **5**; Figure S9: IR spectrum: compound **5**; Figure S10: (a) ^13^H NMR spectrum (101 MHz, DMSO-*d_6_*): compound **6**, (b) ^1^H NMR spectrum (400 MHz, DMSO-*d_6_*): compound **6**; Figure S11: EI-MS spectrum: compound **6**; Figure S12: IR spectrum: compound **6**; Figure S13: (a) ^13^H NMR spectrum (101 MHz, DMSO-*d_6_*): compound **7**, (b) ^1^H NMR spectrum (400 MHz, DMSO-*d_6_*): compound **7**; Figure S14: EI-MS spectrum: compound **7**; Figure S15: IR spectrum: compound **7**; Figure S16: (a) ^13^C NMR spectrum (101 MHz, DMSO-*d_6_*): compound **8**, (b) ^1^H NMR spectrum (400 MHz, DMSO-*d_6_*):compound **8**; Figure S17: EI-MS spectrum: compound **8**; Figure S18: IR spectrum: compound **8**; Figure S19: (a) ^13^C NMR spectrum (101 MHz, DMSO-*d_6_*): compound **9**, (b) ^1^H NMR spectrum (400 MHz, DMSO-*d_6_*): compound **9**; Figure S20: EI-MS spectrum: compound **9**; Figure S21: IR spectrum: compound **9**; Figure S22: (a) ^13^C NMR spectrum (101 MHz, DMSO-*d_6_*): compound **10**, (b) ^1^H NMR spectrum (400 MHz, DMSO-*d_6_*):compound **10**; Figure S23: EI-MS spectrum: compound **10**; Figure S24: IR spectrum: compound **10**; Figure S25: (a) ^13^C NMR spectrum (101 MHz, DMSO-*d_6_*): compound **11**, (b) ^1^H NMR spectrum (400 MHz, DMSO-*d_6_*): compound **11**; Figure S26: EI-MS spectrum: compound **11**; Figure S27: IR spectrum: compound **11;** Table S1: Hydrogen bond parameters; Table S2: Crystal data and structure refinement parameters for selected gramine derivatives.

## Sample Availability

Samples of the compounds are not available from the authors.

## References

[1] Al-Mulla A. (2017). A Review: Biological Importance of Heterocyclic Compounds. Der Pharma Chem..

[2] Dorababu A. (2020). Indole—A Promising Pharmacophore in Recent Antiviral Drug Discovery. RSC Med. Chem..

[3] Dadashpour S., Emami S. (2018). Indole in the Target-Based Design of Anticancer Agents: A Versatile Scaffold with Diverse Mechanisms. Eur. J. Med. Chem..

[4] Song F., Li Z., Bian Y., Huo X., Fang J., Shao L., Zhou M. (2020). Indole/Isatin-Containing Hybrids as Potential Antibacterial Agents. Arch. Pharm..

[5] Chen Y., Li H., Liu J., Zhong R., Li H., Fang S., Liu S., Lin S. (2021). Synthesis and Biological Evaluation of Indole-Based Peptidomimetics as Antibacterial Agents against Gram-Positive Bacteria. Eur. J. Med. Chem..

[6] Kumari A., Singh R.K. (2019). Medicinal Chemistry of Indole Derivatives: Current to Future Therapeutic Prospectives. Bioorg. Chem..

[7] Singh A.A., Patil M.P., Kang M.-J., Niyonizigiye I., Kim G.-D. (2021). Biomedical Application of Indole-3-Carbinol: A Mini-Review. Phytochem. Lett..

[8] Slominski A., Semak I., Pisarchik A., Sweatman T., Szczesniewski A., Wortsman J. (2002). Conversion of L-Tryptophan to Serotonin and Melatonin in Human Melanoma Cells. FEBS Lett..

[9] Strawbridge R., Javed R.R., Cave J., Jauhar S., Young A.H. (2022). The Effects of Reserpine on Depression: A Systematic Review. J. Psychopharmacol..

[10] Li G., Hu Y., Li D., Zhang Y., Guo H., Li Y., Chen F., Xu J. (2020). Vincristine-Induced Peripheral Neuropathy: A Mini-Review. NeuroToxicology.

[11] Moran-Santa Maria M.M., Baker N.L., McRae-Clark A.L., Prisciandaro J.J., Brady K.T. (2016). Effects of yohimbine and drug cues on impulsivity and attention in cocaine-dependent men and women and sex-matched controls. Drug Alcohol Depend..

[12] Zhang X.-H., Guo Q., Wang H.-Y., Li Y.-H., Khamis M.Y., Ma L.-Y., Wang B., Liu H.-M. (2021). Gramine-Based Structure Optimization to Enhance Anti-Gastric Cancer Activity. Bioorg. Chem..

[13] Wei Y., Shi L., Wang K., Liu M., Yang Q., Yang Z., Ke S. (2014). Discovery of Gramine Derivatives That Inhibit the Early Stage of EV71 Replication in Vitro. Molecules.

[14] Süzen S., Khan M.T.H. (2007). Antioxidant Activities of Synthetic Indole Derivatives and Possible Activity Mechanisms. Bioactive Heterocycles V.

[15] Kumar J., Kumar N., Sati N., Hota P.K. (2020). Antioxidant Properties of Ethenyl Indole: DPPH Assay and TDDFT Studies. New J. Chem..

[16] Silveira C.C., Mendes S.R., Soares J.R., Victoria F.N., Martinez D.M., Savegnago L. (2013). Synthesis and Antioxidant Activity of New C-3 Sulfenyl Indoles. Tetrahedron Lett..

[17] Kanwal, Khan K.M., Chigurupati S., Ali F., Younus M., Aldubayan M., Wadood A., Khan H., Taha M., Perveen S. (2021). Indole-3-Acetamides: As Potential Antihyperglycemic and Antioxidant Agents; Synthesis, In Vitro α-Amylase Inhibitory Activity, Structure–Activity Relationship, and In Silico Studies. ACS Omega.

[18] Jasiewicz B., Kozanecka-Okupnik W., Przygodzki M., Warżajtis B., Rychlewska U., Pospieszny T., Mrówczyńska L. (2021). Synthesis, Antioxidant and Cytoprotective Activity Evaluation of C-3 Substituted Indole Derivatives. Sci. Rep..

[19] Kozanecka-Okupnik W., Jasiewicz B., Pospieszny T., Jastrząb R., Skrobańska M., Mrówczyńska L. (2018). Spectroscopy, Molecular Modeling and Anti-Oxidant Activity Studies on Novel Conjugates Containing Indole and Uracil Moiety. J. Mol. Struct..

[20] Kozanecka-Okupnik W., Sierakowska A., Berdzik N., Kowalczyk I., Mrówczyńska L., Jasiewicz B. (2022). New Triazole-Bearing Gramine Derivatives—Synthesis, Structural Analysis and Protective Effect against Oxidative Haemolysis. Nat. Prod. Res..

[21] Rosemeyer H. (2004). The Chemodiversity of Purine as a Constituent of Natural Products. Chem. Biodivers..

[22] Verma A., Joshi S., Singh D. (2013). Imidazole: Having Versatile Biological Activities. J. Chem..

[23] Alghamdi S.S., Suliman R.S., Almutairi K., Kahtani K., Aljatli D. (2021). Imidazole as a Promising Medicinal Scaffold: Current Status and Future Direction. Drug Des. Devel. Ther..

[24] Bozdag M., Supuran C.T., Esposito D., Angeli A., Carta F., Monti S.M., De Simone G., Alterio V. (2020). 2-Mercaptobenzoxazoles: A Class of Carbonic Anhydrase Inhibitors with a Novel Binding Mode to the Enzyme Active Site. Chem. Commun. Camb. Engl..

[25] Azam M.A., Suresh B. (2012). Biological Activities of 2-Mercaptobenzothiazole Derivatives: A Review. Sci. Pharm..

[26] Safak C., Simsek R., Erol K., Vural K. (1996). Analgesic and Antiinflammatory Effects of Some 2-Mercaptobenzoxazole Derivatives. Die Pharm..

[27] Staniszewska M., Kuryk Ł., Gryciuk A., Kawalec J., Rogalska M., Baran J., Łukowska-Chojnacka E., Kowalkowska A. (2021). In Vitro Anti-Candida Activity and Action Mode of Benzoxazole Derivatives. Molecules.

[28] Mir F., Shafi S., Zaman M.S., Kalia N.P., Rajput V.S., Mulakayala C., Mulakayala N., Khan I.A., Alam M.S. (2014). Sulfur Rich 2-Mercaptobenzothiazole and 1,2,3-Triazole Conjugates as Novel Antitubercular Agents. Eur. J. Med. Chem..

[29] Karaca Ş., Osmaniye D., Sağlık B.N., Levent S., Ilgın S., Özkay Y., Karaburun A.Ç., Kaplancıklı Z.A., Gundogdu-Karaburun N. (2022). Synthesis of Novel Benzothiazole Derivatives and Investigation of Their Enzyme Inhibitory Effects against Alzheimer’s Disease. RSC Adv..

[30] Decker M., Decker M. (2017). Introduction. Design of Hybrid Molecules for Drug Development.

[31] Singla R., Gupta K.B., Upadhyay S., Dhiman M., Jaitak V. (2018). Design, Synthesis and Biological Evaluation of Novel Indole-Benzimidazole Hybrids Targeting Estrogen Receptor Alpha (ER-α). Eur. J. Med. Chem..

[32] Wang R., Shi H.-F., Zhao J.-F., He Y.-P., Zhang H.-B., Liu J.-P. (2013). Design, Synthesis and Aromatase Inhibitory Activities of Novel Indole-Imidazole Derivatives. Bioorg. Med. Chem. Lett..

[33] Naureen S., Ijaz F., Nazeer A., Chaudhry F., Munawar M.A., Khan M.A. (2017). Facile, Eco-Friendly, One-Pot Protocol for the Synthesis of Indole-Imidazole Derivatives Catalyzed by Amino Acids. Synth. Commun..

[34] Hogendorf A.S., Hogendorf A., Popiołek-Barczyk K., Ciechanowska A., Mika J., Satała G., Walczak M., Latacz G., Handzlik J., Kieć-Kononowicz K. (2019). Fluorinated Indole-Imidazole Conjugates: Selective Orally Bioavailable 5-HT7 Receptor Low-Basicity Agonists, Potential Neuropathic Painkillers. Eur. J. Med. Chem..

[35] Naureen S., Chaudhry F., Munawar M.A., Ashraf M., Hamid S., Khan M.A. (2018). Biological Evaluation of New Imidazole Derivatives Tethered with Indole Moiety as Potent α-Glucosidase Inhibitors. Bioorg. Chem..

[36] James D.A., Koya K., Li H., Chen S., Xia Z., Ying W., Wu Y., Sun L. (2006). Conjugated Indole-Imidazole Derivatives Displaying Cytotoxicity against Multidrug Resistant Cancer Cell Lines. Bioorg. Med. Chem. Lett..

[37] Li Z.-Z., Tangadanchu V.K.R., Battini N., Bheemanaboina R.R.Y., Zang Z.-L., Zhang S.-L., Zhou C.-H. (2019). Indole-Nitroimidazole Conjugates as Efficient Manipulators to Decrease the Genes Expression of Methicillin-Resistant Staphylococcus Aureus. Eur. J. Med. Chem..

[38] Pillaiyar T., Uzair M., Ullah S., Schnakenburg G., Müller C.E. (2019). Decarboxylative Coupling Reaction of 2-(1H-Indol-3-Yl)Acetic Acids with Indole, Azaindole, Benzimidazole and Indazole Derivatives. Adv. Synth. Catal..

[39] Decods G., Wakselman M. (1968). Condensations thermiques de la gramine avec les polyphénols, l’imidazole et ses derives. Comptes Rendus Hebd. Séances Académie Sci..

[40] Podsiedlik M., Markowicz-Piasecka M., Sikora J. (2020). Erythrocytes as model cells for biocompatibility assessment, cytotoxicity screening of xenobiotics and drug delivery. Chem. Biol. Interact..

[41] Liguori I., Russo G., Curcio F., Bulli G., Aran L., Della-Morte D., Gargiulo G., Testa G., Cacciatore F., Bonaduce D. (2018). Oxidative stress, aging, and diseases. Clin. Interv. Aging..

[42] Gulcin I., Alwasel S.H. (2022). Metal ions, metal chelators and metal chelating assay as antioxidant method. Processes.

[43] Zhu M., Zhu Q., Yang Z., Liang Z. (2021). Clinical Characteristics of Patients with *Micrococcus luteus* Bloodstream Infection in a Chinese Tertiary-Care Hospital. Pol. J. Microbiol..

[44] Necel A., Bloch S., Topka-Bielecka G., Janiszewska A., Łukasiak A., Nejman-Faleńczyk B., Węgrzyn G. (2022). Synergistic Effects of Bacteriophage vB_Eco4-M7 and Selected Antibiotics on the Biofilm Formed by Shiga Toxin-Producing *Escherichia coli*. Antibiotics.

[45] Wołejko E., Wydro U., Jabłońska-Trypuć A., Butarewicz A., Łoboda T. (2018). Pseudomonas fluoresces occurrence in soil after fertilization with sewage sludge. Ekon. I Sr. —Econ. Environ..

[46] Kozicki M., Wiejak A., Piasecki M., Abram A. (2019). Identification of MVOCs Produced by Coniophora puteana and Poria placenta Growing on WPC Boards by Using Subtraction Mass Spectra. Int. J. Environ. Res. Public Health.

[47] Daina A., Michielin O., Zoete V. (2017). SwissADME: A Free Web Tool to Evaluate Pharmacokinetics, Drug-Likeness and Medicinal Chemistry Friendliness of Small Molecules. Sci. Rep..

[48] Lipinski C.A. (2004). Lead- and Drug-like Compounds: The Rule-of-Five Revolution. Drug Discov. Today Technol..

[49] Roleira F.M.F., Siquet C., Orrù E., Garrido E.M., Garrido J., Milhazes N., Podda G., Paiva-Martins F., Reis S., Carvalho R.A. (2010). Lipophilic phenolic antioxidants: Correlation between antioxidant profile, partition coefficients and redox properties. Bioorg. Med. Chem..

[50] Veber D.F., Johnson S.R., Cheng H.Y., Smith B.R., Ward K.W., Kopple K.D. (2002). Molecular properties that influence the oral bioavailability of drug candidates. J. Med. Chem..

[51] Daina A., Zoete V.A. (2016). BOILED-Egg to Predict Gastrointestinal Absorption and Brain Penetration of Small Molecules. Chem. Med. Chem..

[52] Savjani K.T., Gajjar A.K., Savjani J.K. (2012). Drug Solubility: Importance and Enhancement Techniques. ISRN Pharm..

[53] CrysAlisPro (2014). Agilent Technologies, Version 1.171.37.33.

[54] Sheldrick G.M. (2015). *SHELXT*—Integrated space-group and crystal-structure determination. Acta Crystallogr. Sect. A Found. Adv..

[55] Sheldrick G.M. (2015). Crystal structure refinement with *SHELXL*, *Acta Crystallogr*. Sect. C Struct. Chem..

[56] Bruno I.J., Cole J.C., Edgington P.R., Kessler M., Macrae C.F., McCabe P., Pearson J., Taylor R. (2002). New software for searching the Cambridge Structural Database and visualizing crystal structures. Acta Crystallogr. Sect. B Struct. Sci..

[57] Mrówczyńska L., Hagerstrand H. (2009). Platelet-activating factor interaction with the human erythrocyte membrane. J. Biochem. Mol. Toxicol..

[58] Ważny J., Thornton J.D. (1986). Comparative Laboratory Testing of Strains of the Dry Rot Fungus *Serpula lacrymans* (Schum. ex Fr.) S.F. Gray. II. The Action of Some Wood Preservatives in Agar Media. Holzforschung.

