# Supplementary material for: Indole Derivatives Bearing Imidazole, Benzothiazole-2-Thione or Benzoxazole-2-Thione Moieties—Synthesis, Structure and Evaluation of Their Cytoprotective, Antioxidant, Antibacterial and Fungicidal Activities
Source: Molecules. 2023 Jan 10;28(2):708. doi: 10.3390/molecules28020708 (PMC9867442; doi:10.3390/molecules28020708)
Supplement: Supplementary file 1 [file molecules-28-00708-s001.zip › molecules-2104568-supplementary.pdf]

**Supplementary data**

# Indole derivatives bearing imidazole, benzothiazole-2-thione and benzoxazole-2-thione moieties – synthesis, structure and evaluation of their cytoprotective, antioxidant, antibacterial and fungicidal activities

Beata Jasiewicz[1], Karolina Babijczuk[1], Beata Warżajtis[2], Urszula Rychlewska[2], Justyna Starzyk[3], Grzegorz Cofta[4] and Lucyna Mrówczyńska[5]

[1]*Department of Bioactive Products, Faculty of Chemistry, Adam Mickiewicz University, Uniwersytetu Poznańskiego 8, 61-614 Poznań, Poland,*
[2]*Department of Crystallography, Faculty of Chemistry, Adam Mickiewicz University, Uniwersytetu Poznańskiego 8, 61-614 Poznań, Poland,*
[3]*Department of Soil Science and Microbiology, Faculty of Agronomy, Horticulture, and Bioengineering, University of Life Science, Szydłowska 50, 60-656 Poznań, Poland,*
[4]*Department of Wood Chemical Technology, Faculty of Forest and Wood Technology, University of Life Science, Wojska Polskiego 28, 60-637 Poznań, Poland,*
[5]*Department of Cell Biology, Faculty of Biology, Adam Mickiewicz University, Uniwersytetu Poznańskiego 6, 61-614 Poznań, Poland,*

## TABLE OF CONTENTS

$^1$H and $^{13}$C NMR spectra of compound **3**……………..…..…………………………………….S3
EI-MS and IR spectra of compound **3**……..…………………………………………………….S4
$^1$H and $^{13}$C NMR spectra of compound 4……………………………………………………….S5
EI-MS and IR spectra of compound **4**……..…………………………………………………….S6
$^1$H and $^{13}$C NMR spectra of compound **5**……………………………………………………….S7
EI-MS and IR spectra of compound **5**……..…………………………………………..……….S8
$^1$H and $^{13}$C NMR spectra of compound **6**…………………………………………………......S9
EI-MS and IR spectra of compound **6**……..………………………………………..…….S10
$^1$H and $^{13}$C NMR spectra of compound **7**……………………………………………..…….S11
EI-MS and IR spectra of compound **7**……..…………………………………………..…….S12
$^1$H and $^{13}$C NMR spectra of compound **8**……………………………………………..…….S13
EI-MS and IR spectra of compound **8**……..……………………….………………….S14
$^1$H and $^{13}$C NMR spectra of compound **9**………………………………………………...S15

EI-MS and IR spectra of compound **9**……………………………………………………..….S16
$^{1}$H and $^{13}$C NMR spectra of compound **10**………………………………………………….S17
EI-MS and IR spectra of compound **10**……………………………………………………….S18
$^{1}$H and $^{13}$C NMR spectra of compound **11**………………………………………………….S19
EI-MS and IR spectra of compound **11**……………………………………………………….S20
Table S1. Hydrogen bond parameters……………………………………………………….S21
Table S2. Crystal data and structure refinement parameters for selected gramine derivatives……………………………………………………………………………..….S22

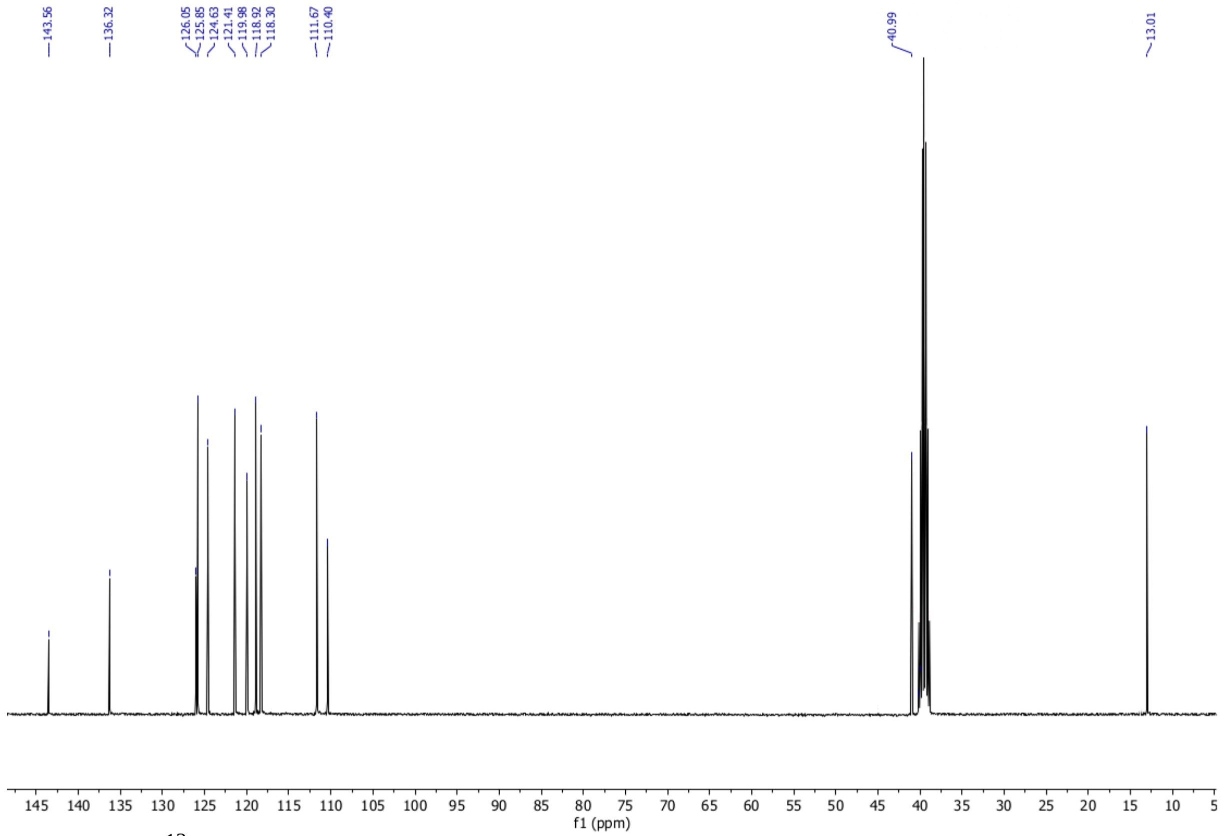


**Figure S1a.** $^{13}$C NMR spectrum of compound **3**

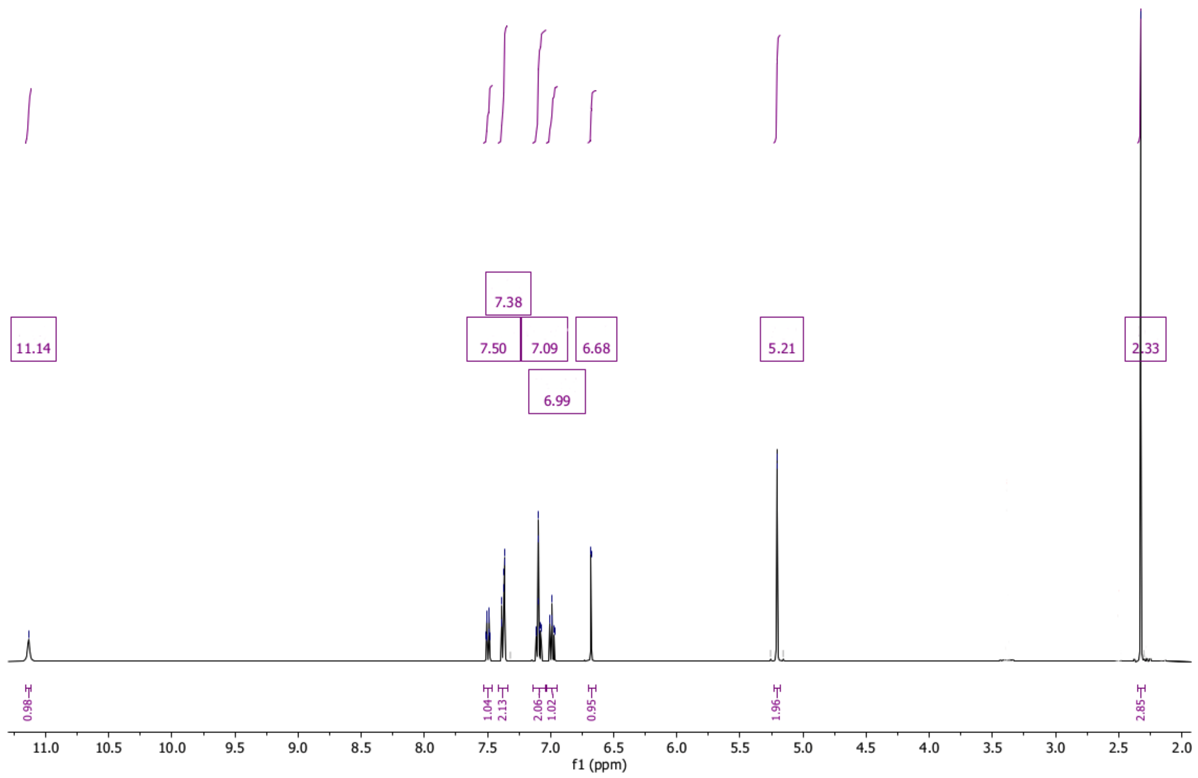


**Figure S1b.** $^{1}$H NMR spectrum of compound **3**

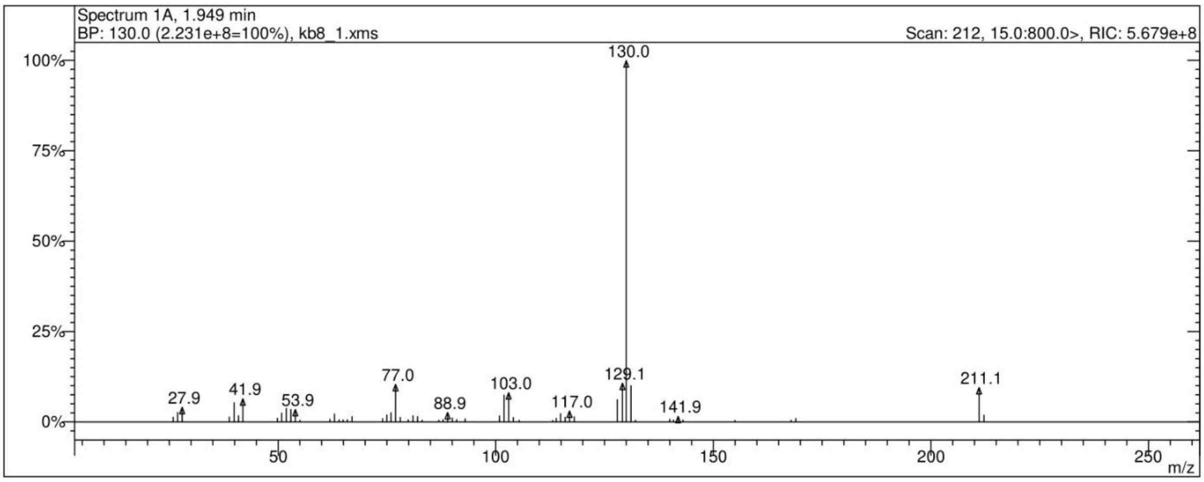


**Figure S2.** EI-MS spectrum of compound **3**

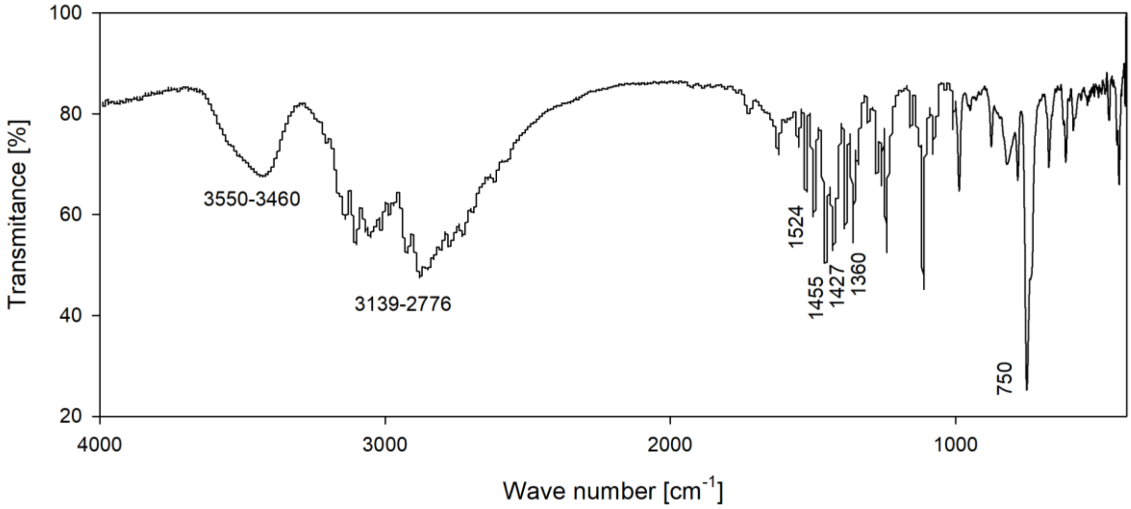


**Figure S3.** IR spectrum of compound **3**

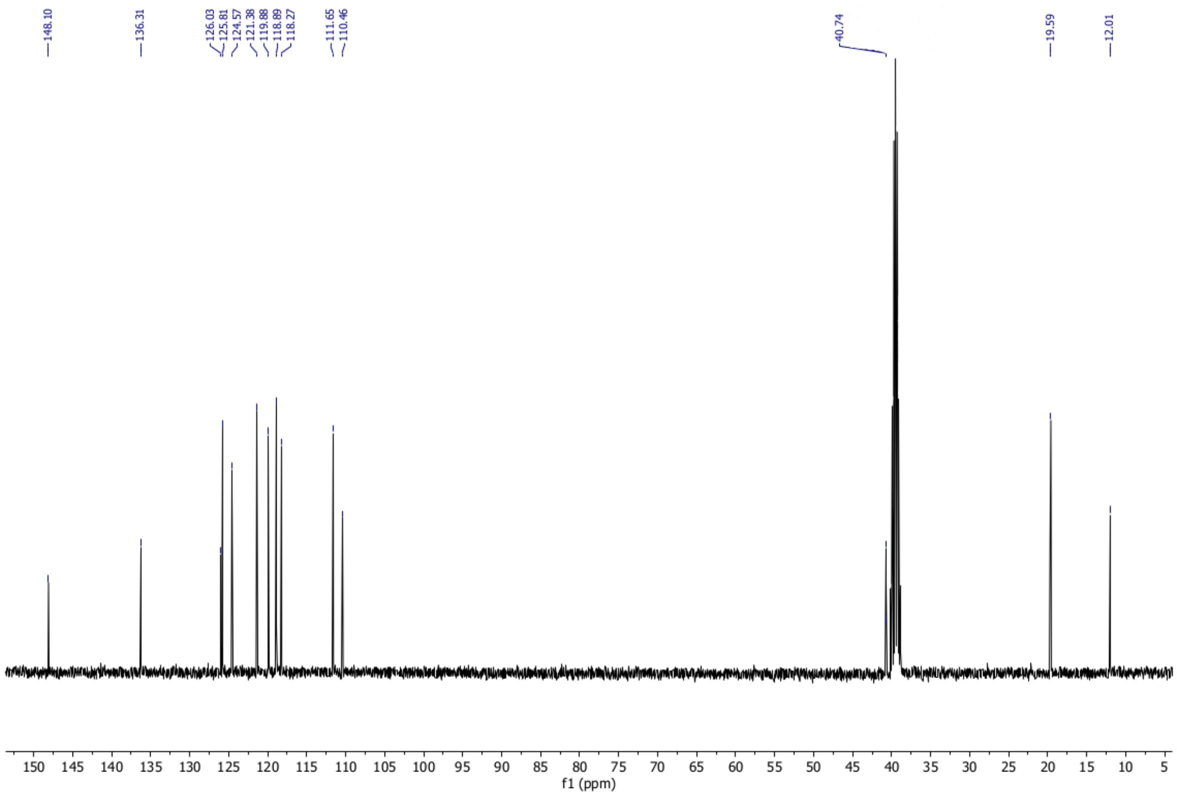


**Figure S4a.** $^{13}$C NMR spectrum of compound **4**

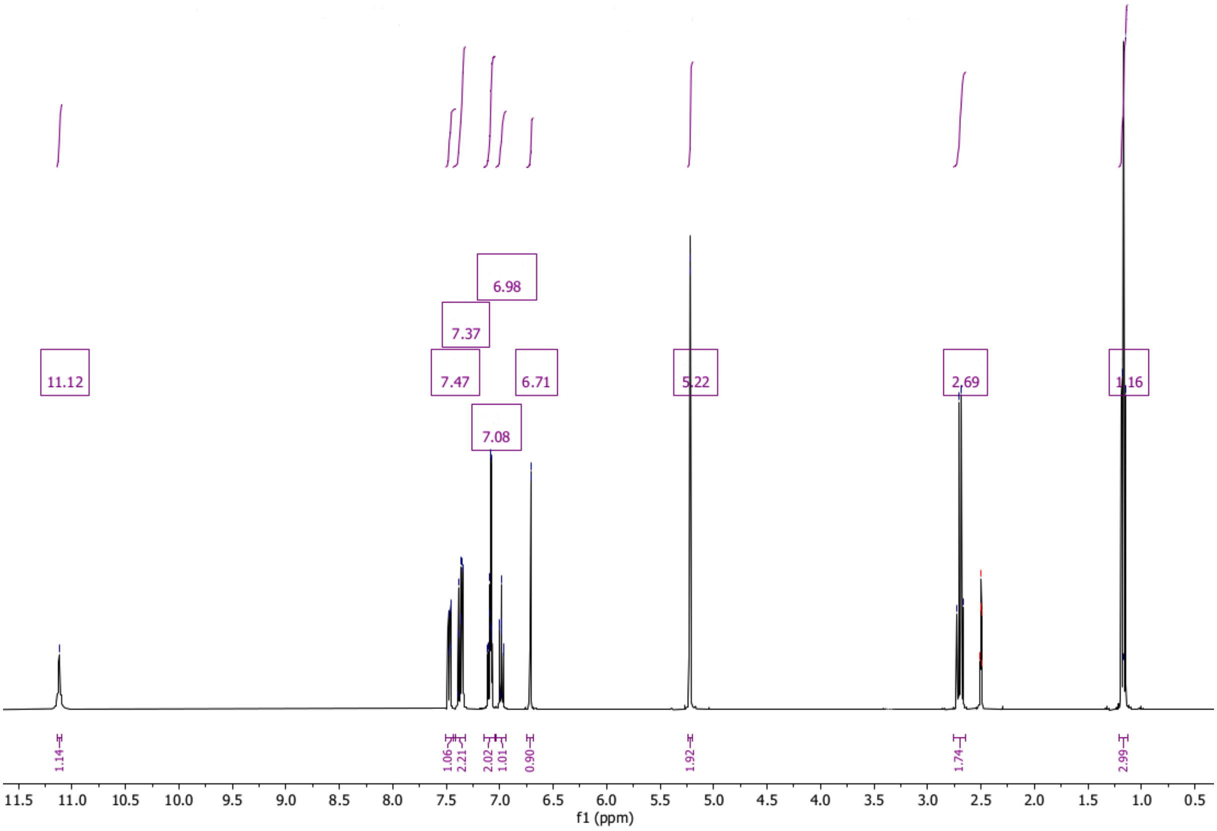


**Figure S4b.** $^{1}$H NMR spectrum of compound **4**

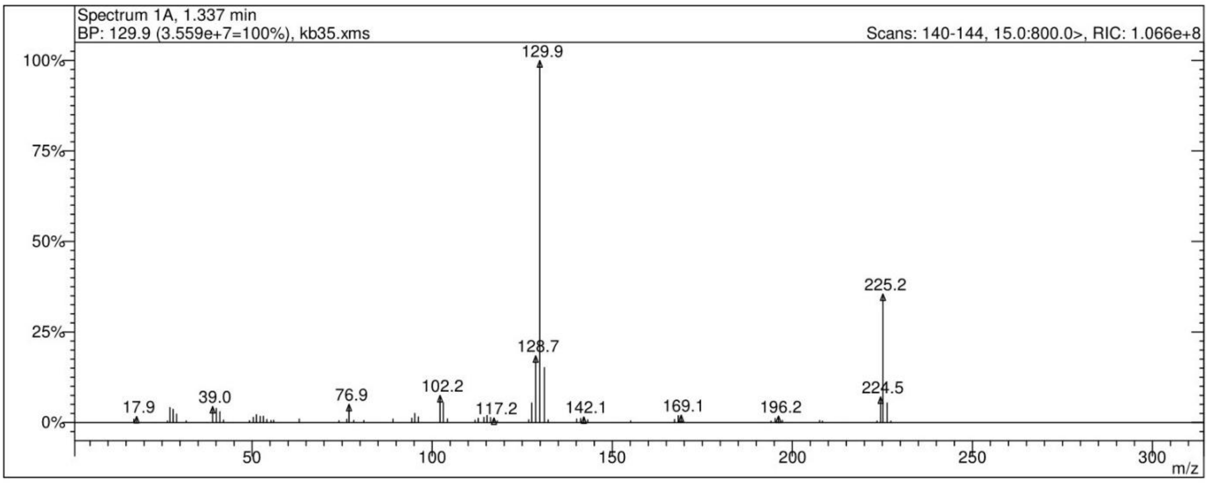


**Figure S5.** EI-MS spectrum of compound **4**

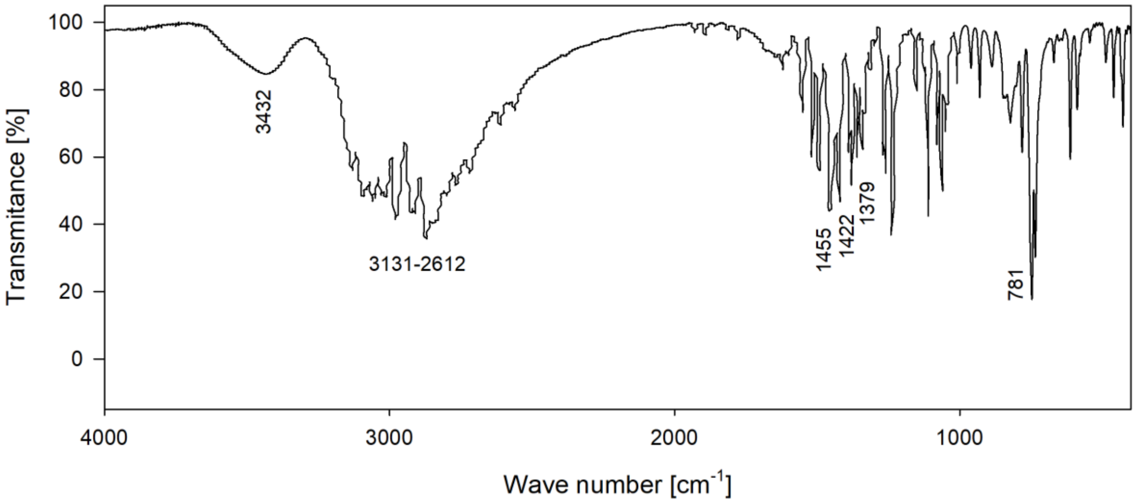


**Figure S6.** FTIR spectrum of compound **4**

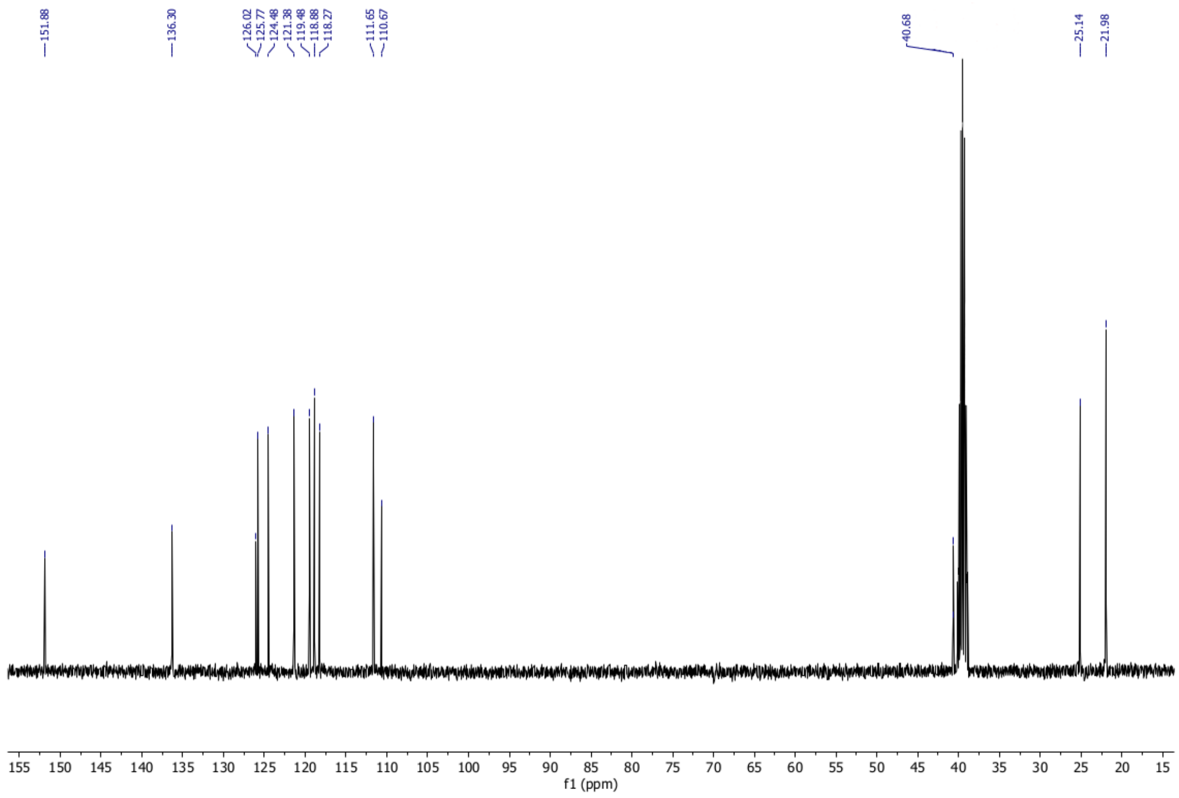


**Figure S7a.** $^{13}$C NMR spectrum of compound **5**

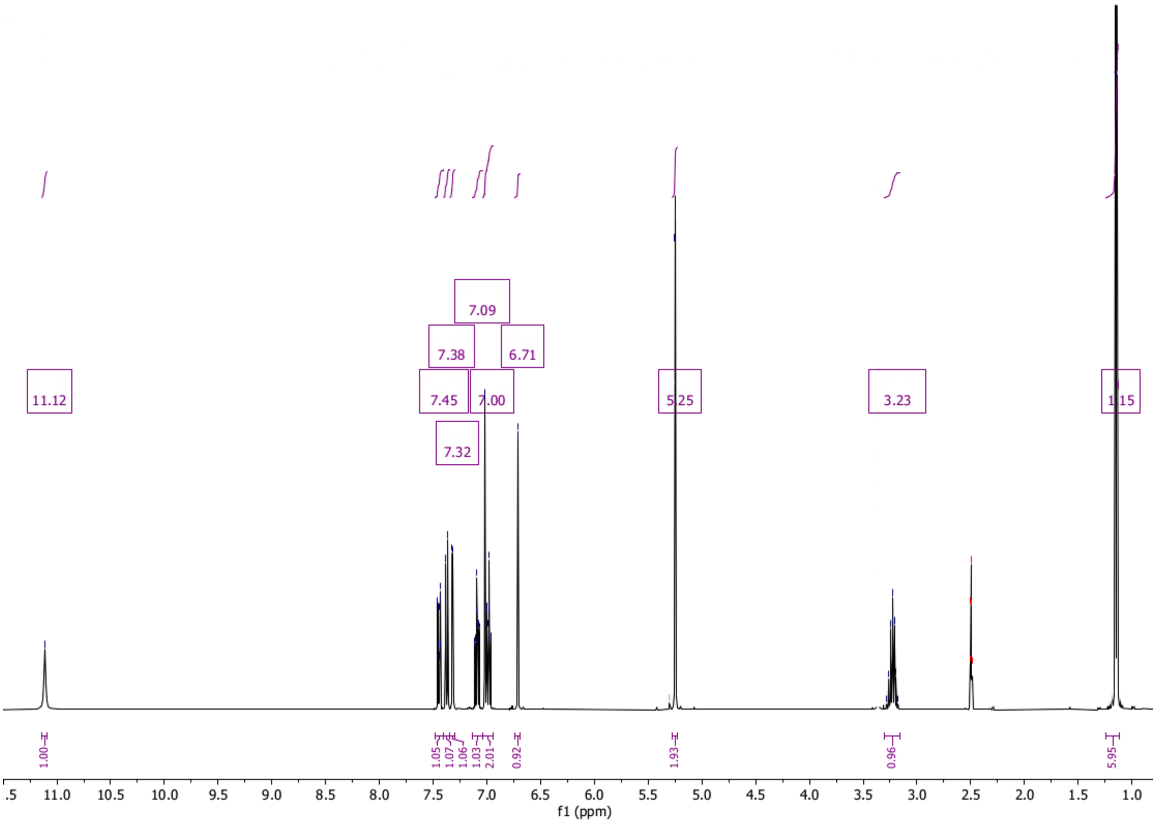


**Figure S7b.** $^{1}$H NMR spectrum of compound **5**

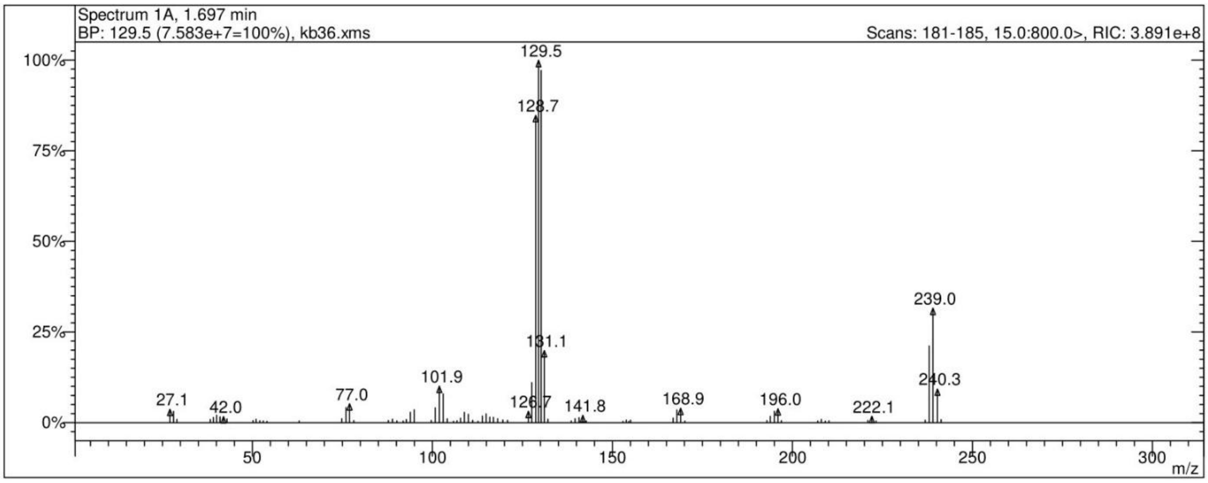


**Figure S8.** EI-MS spectrum of compound **5**

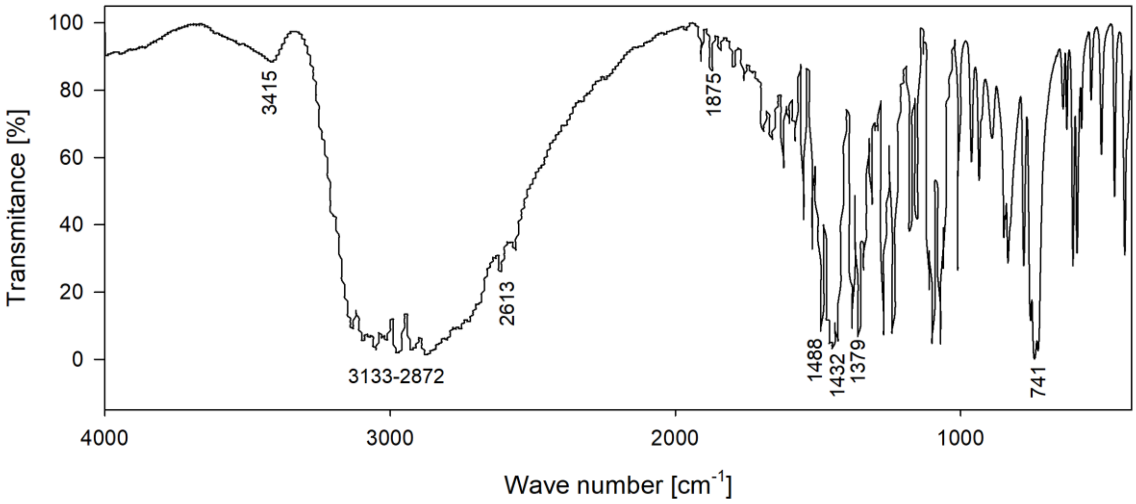


**Figure S9.** IR spectrum of compound **5**

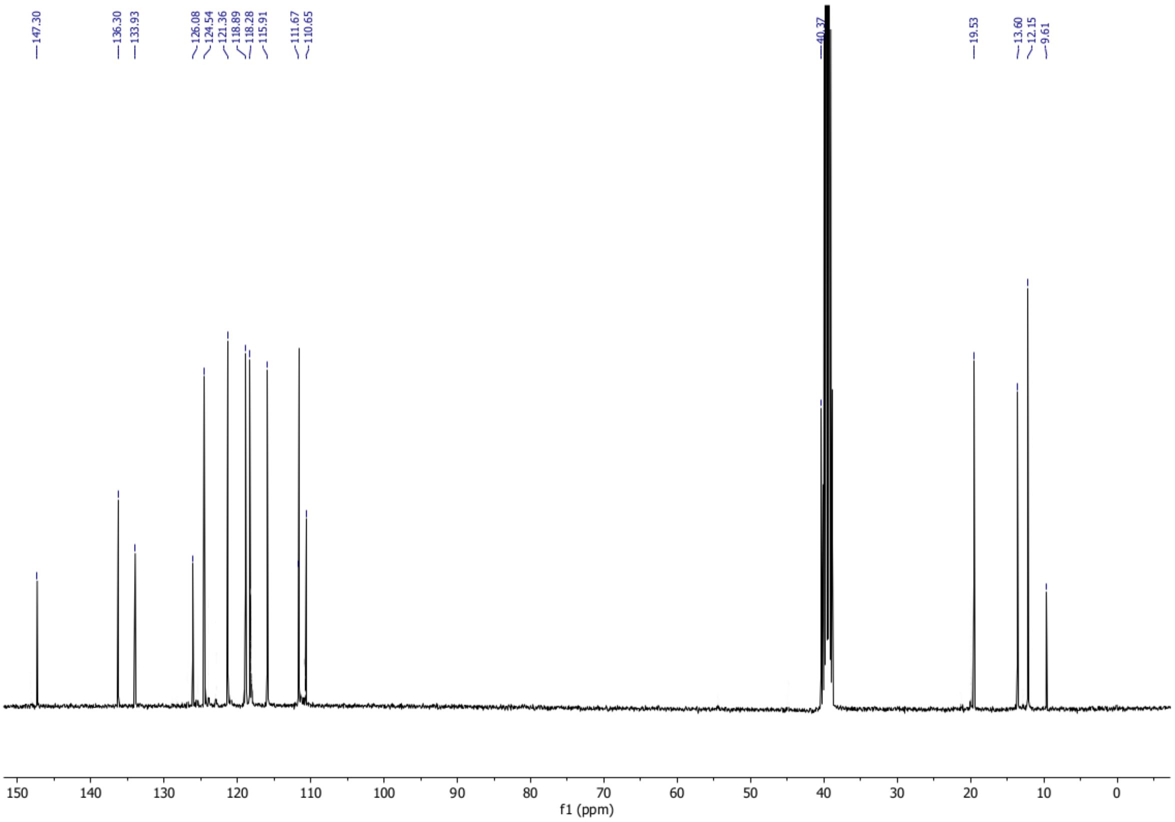


**Figure S10a.** $^{13}$C NMR spectrum of compound **6**

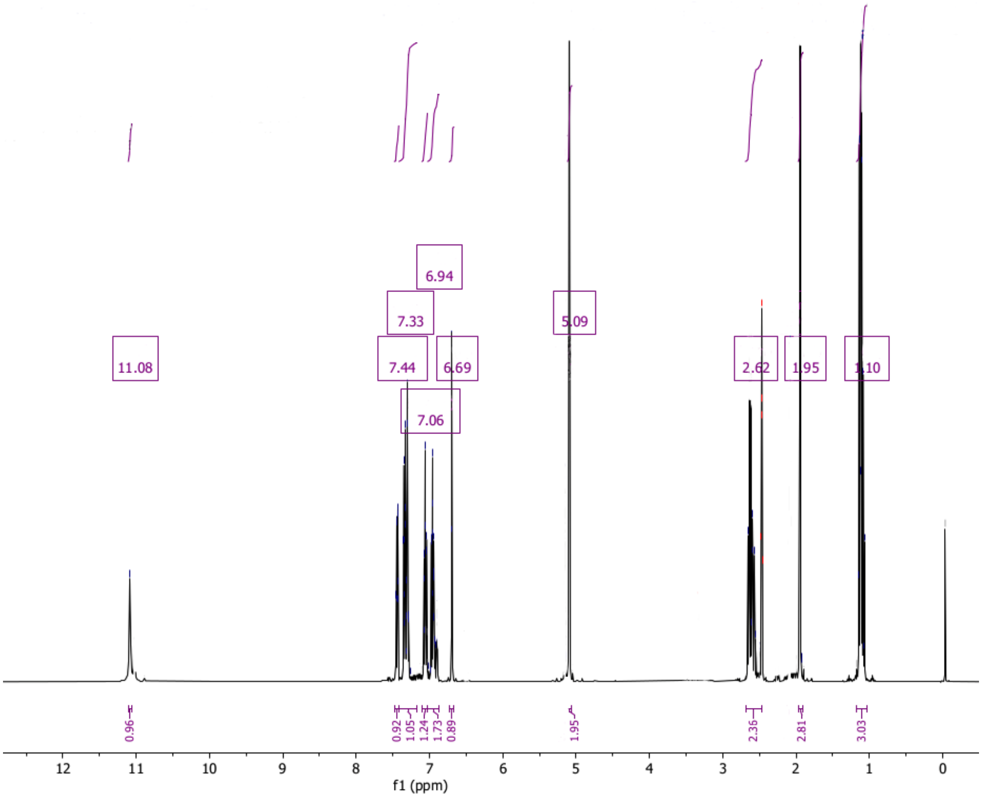


**Figure S10b.** $^{1}$H NMR spectrum of compound **6**

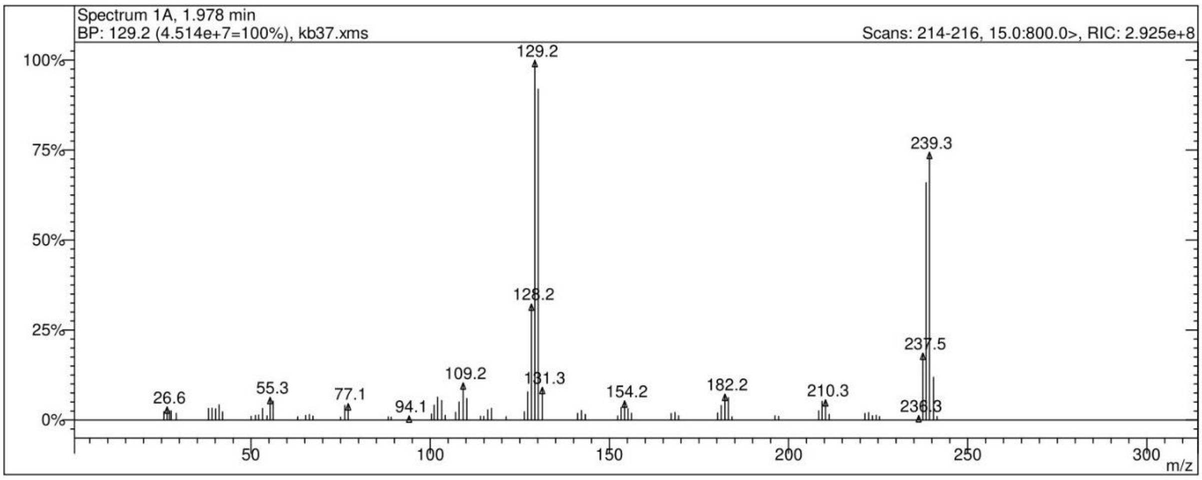


**Figure S11.** EI-MS spectrum of compound **6**

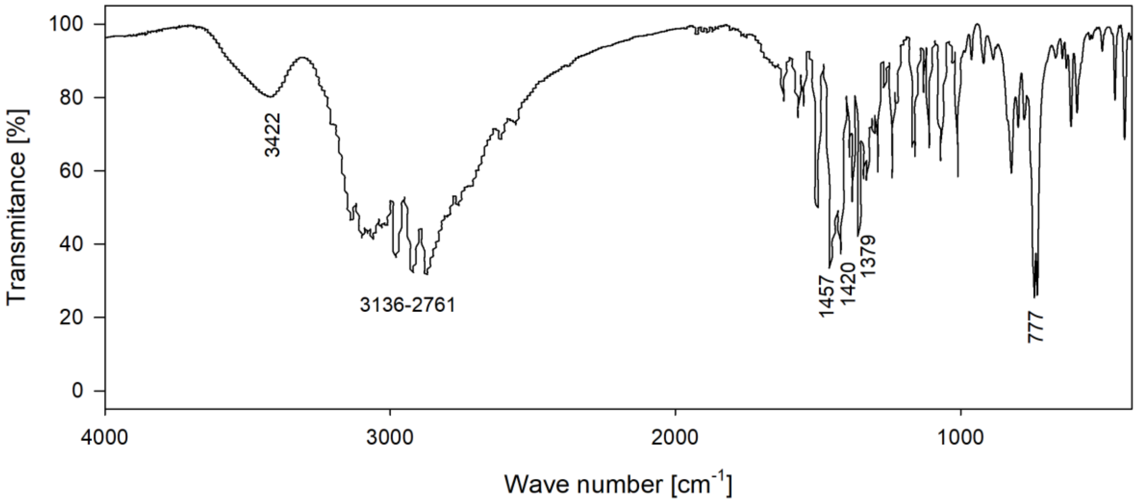


**Figure S12.** IR spectrum of compound **6**

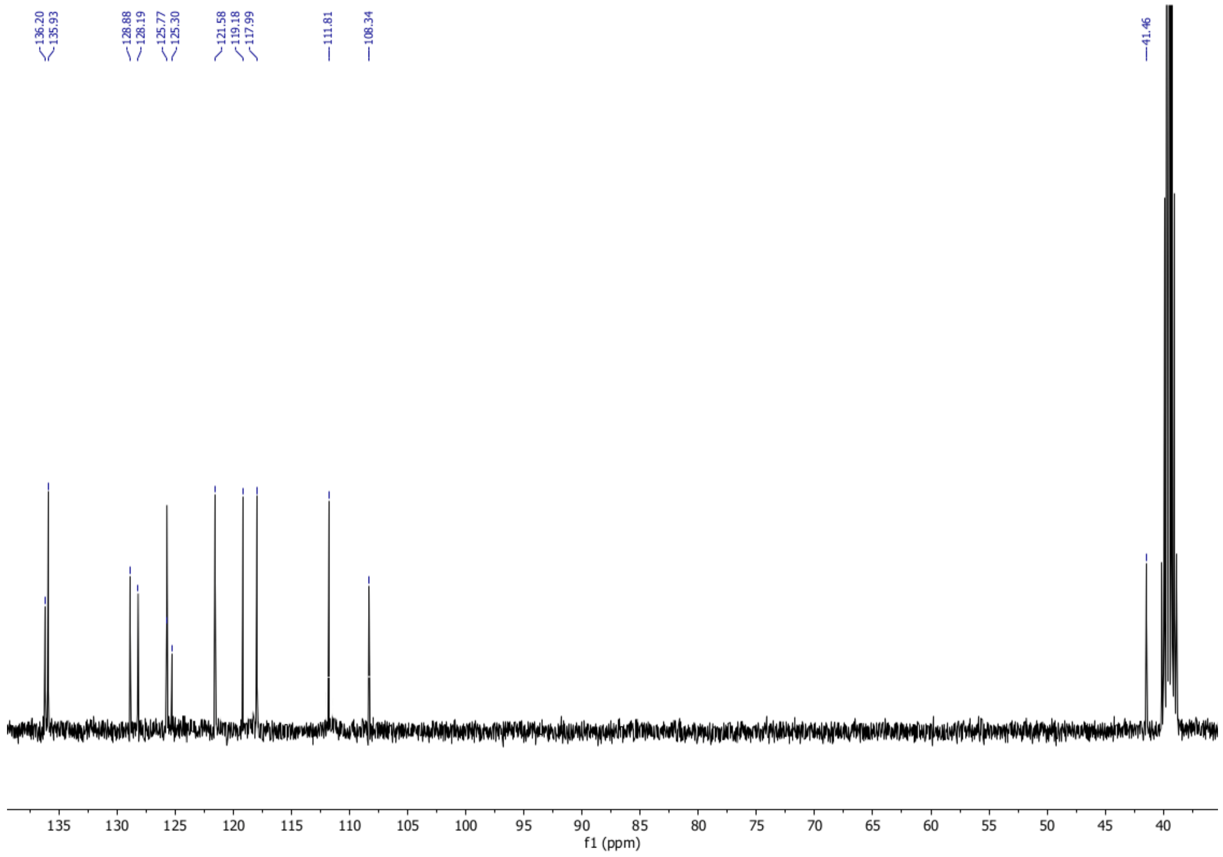


**Figure S13a.** $^{13}$C NMR spectrum of compound **7**

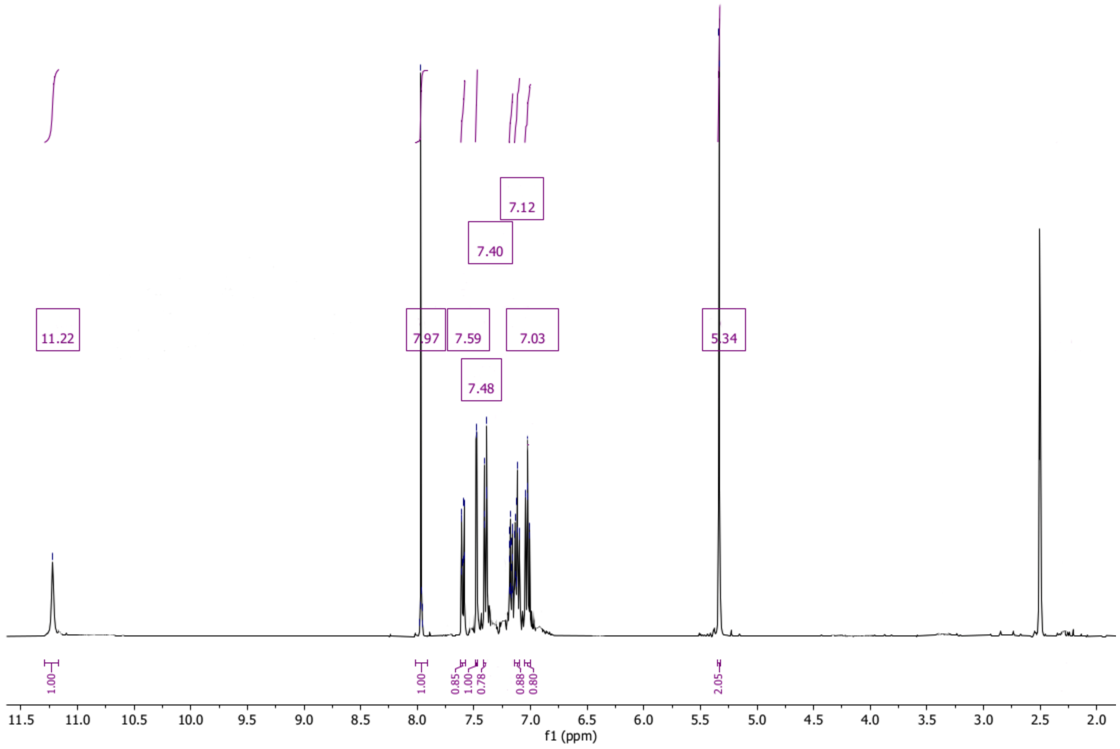


**Figure S13b.** $^{1}$H NMR spectrum of compound **7**

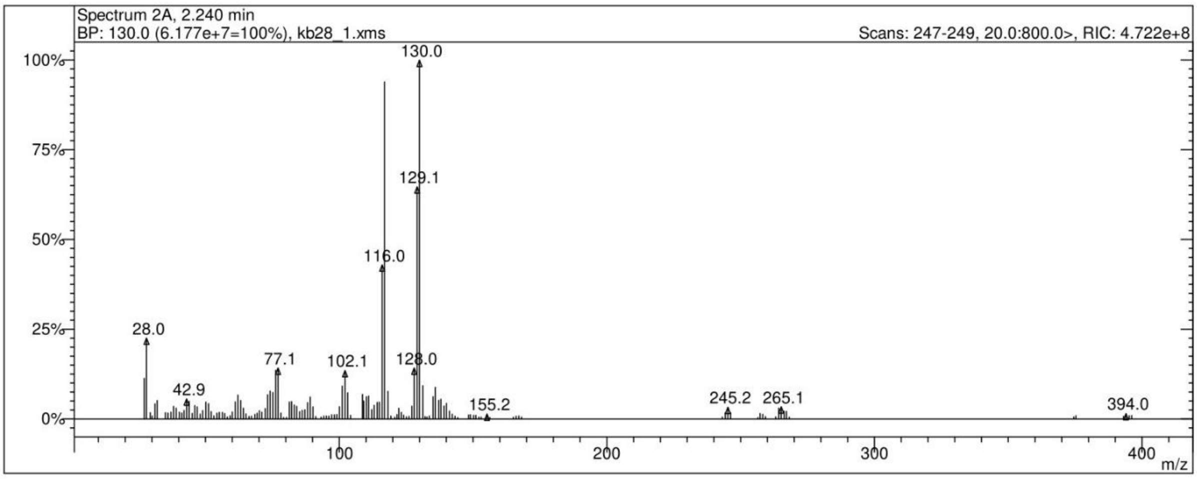


**Figure S14.** EI-MS spectrum of compound **7**

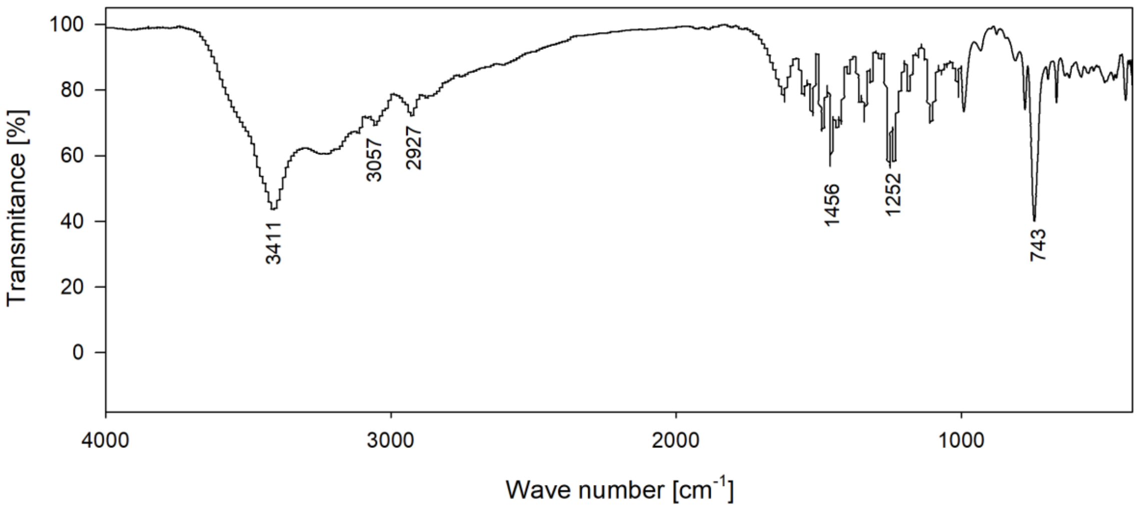


**Figure S15.** IR spectrum of compound **7**

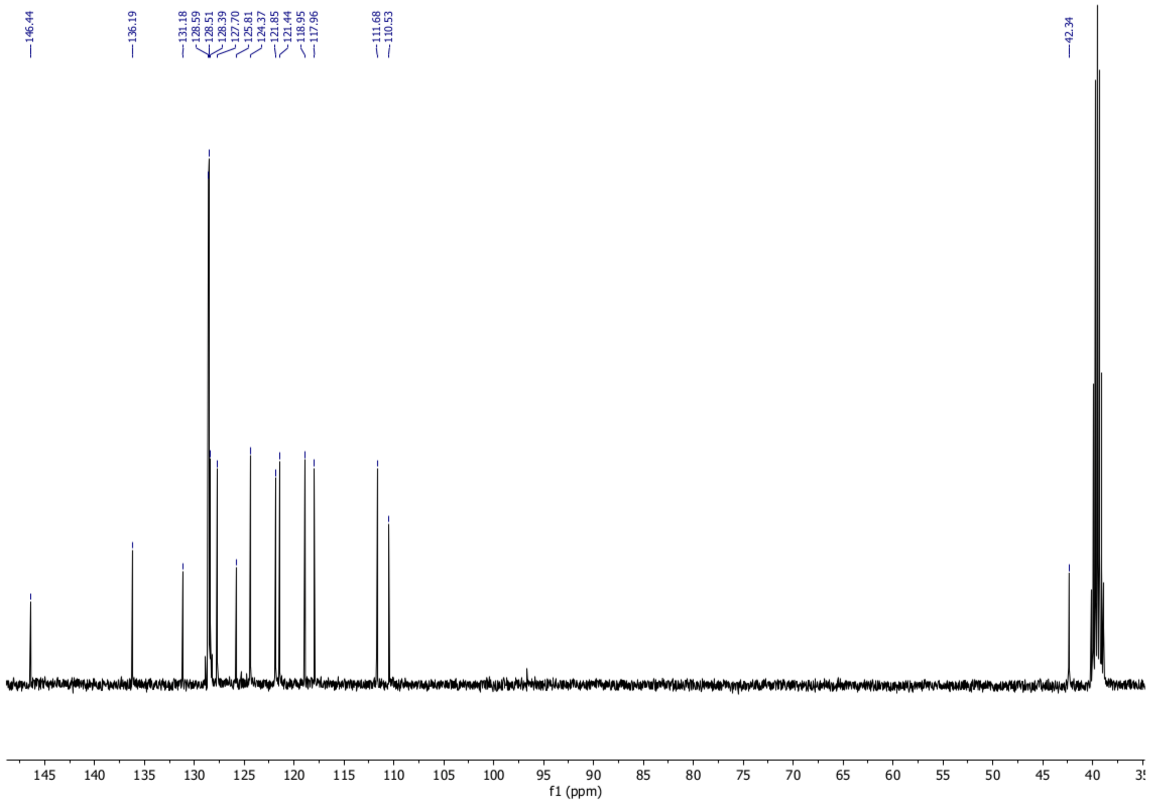


**Figure S16a.** $^{13}C$ NMR spectrum of compound **8**

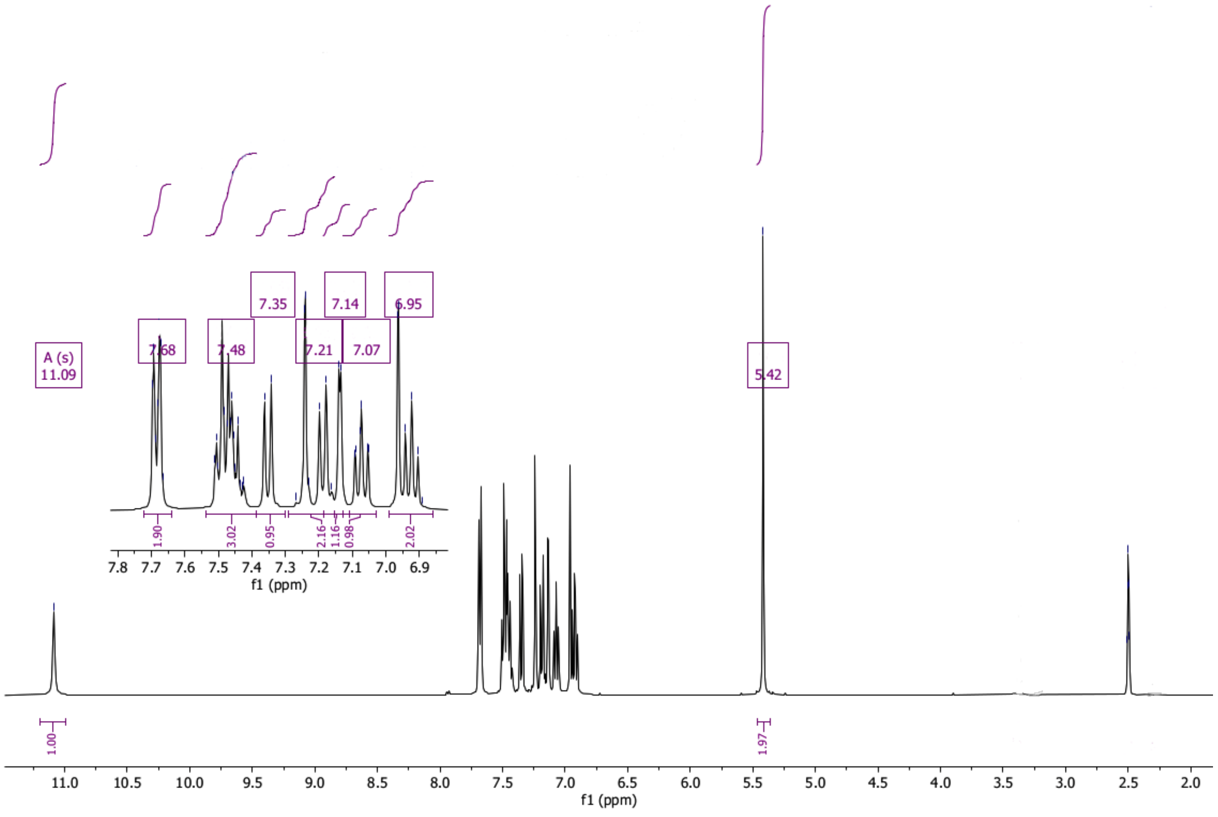


**Figure S16b.** $^{1}H$ NMR spectrum of compound **8**

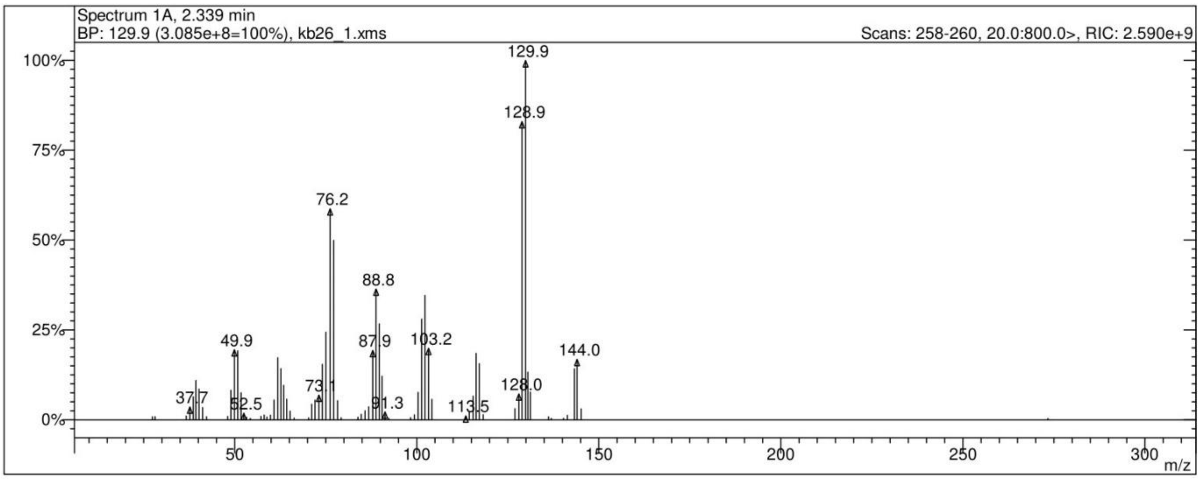


**Figure S17.** EI-MS spectrum of compound **8**

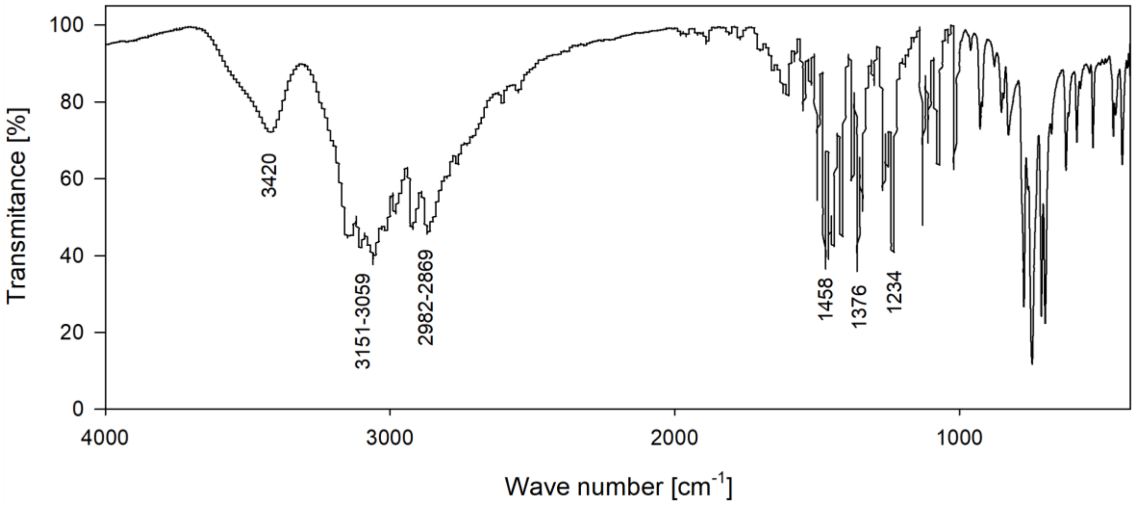


**Figure S18.** IR spectrum of compound **8**

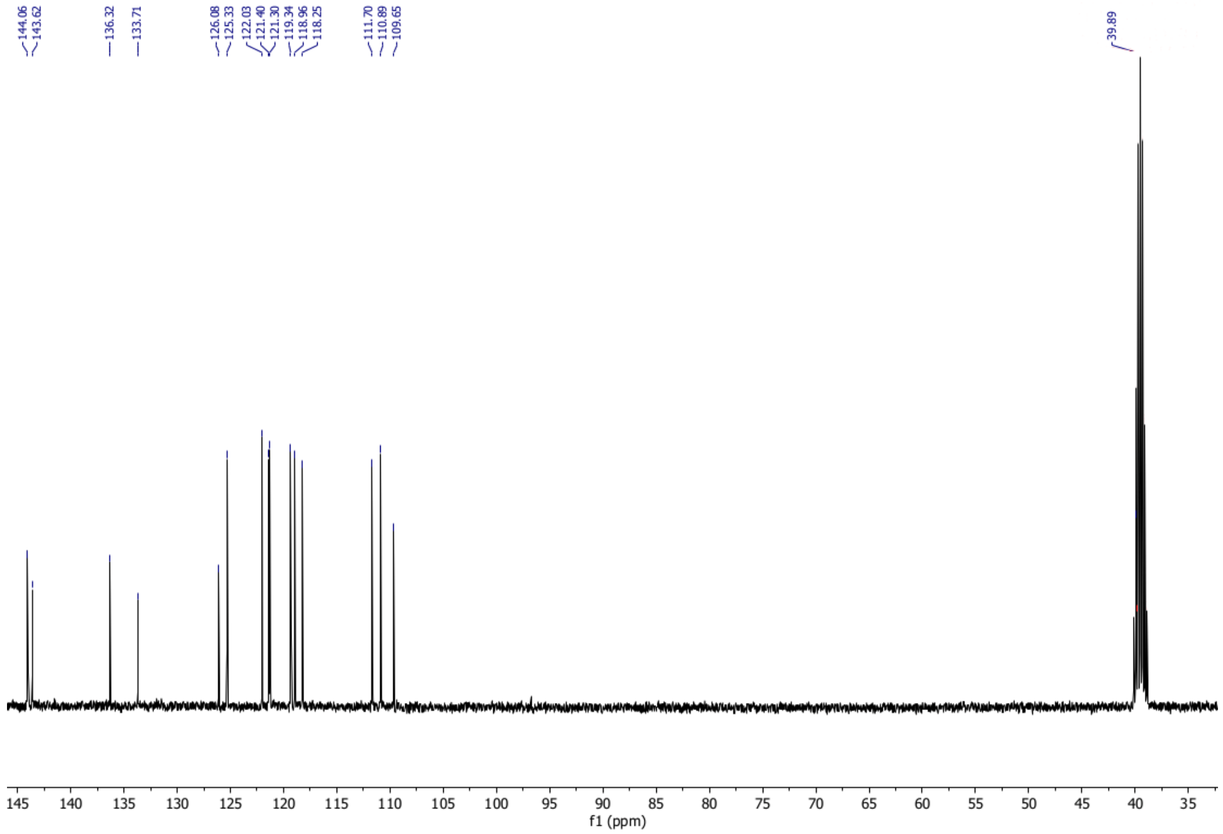


**Figure S19a.** $^{13}$C NMR spectrum of compound **9**

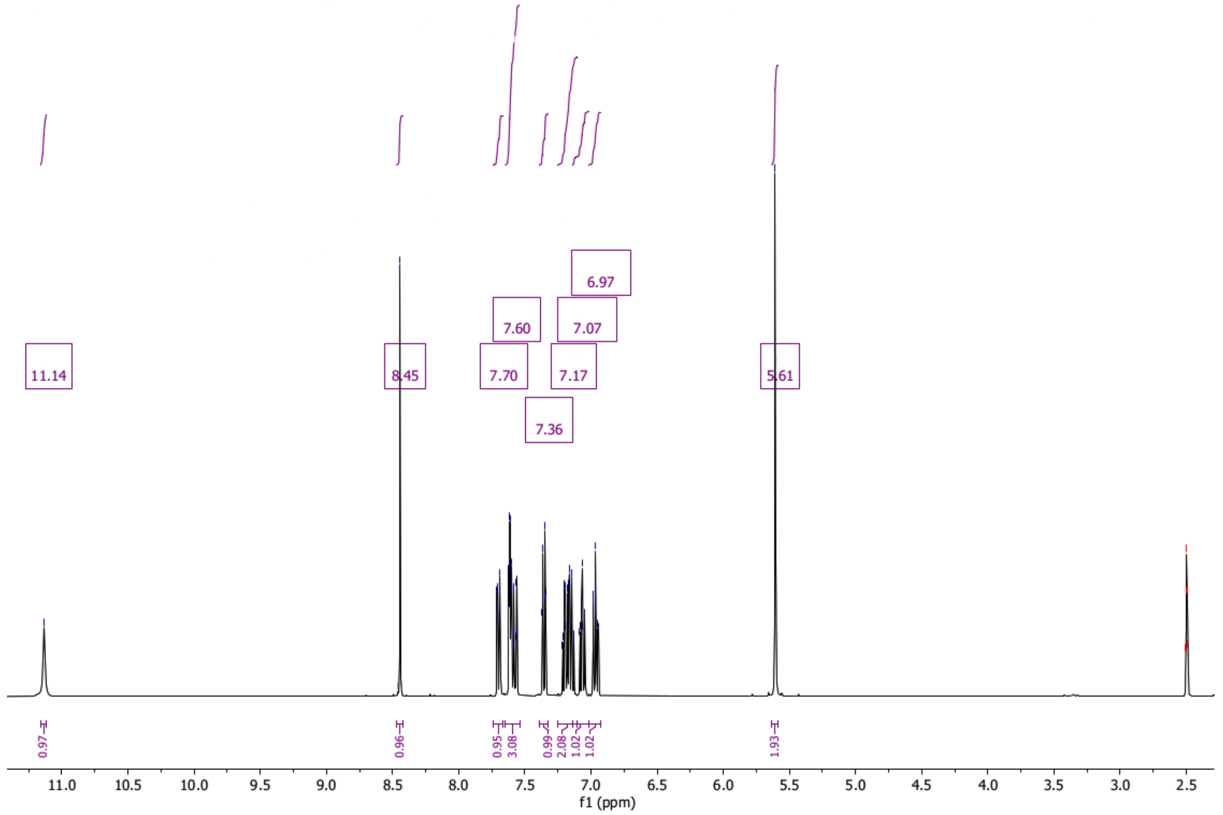


**Figure S19b.** $^{1}$H NMR spectrum of compound **9**

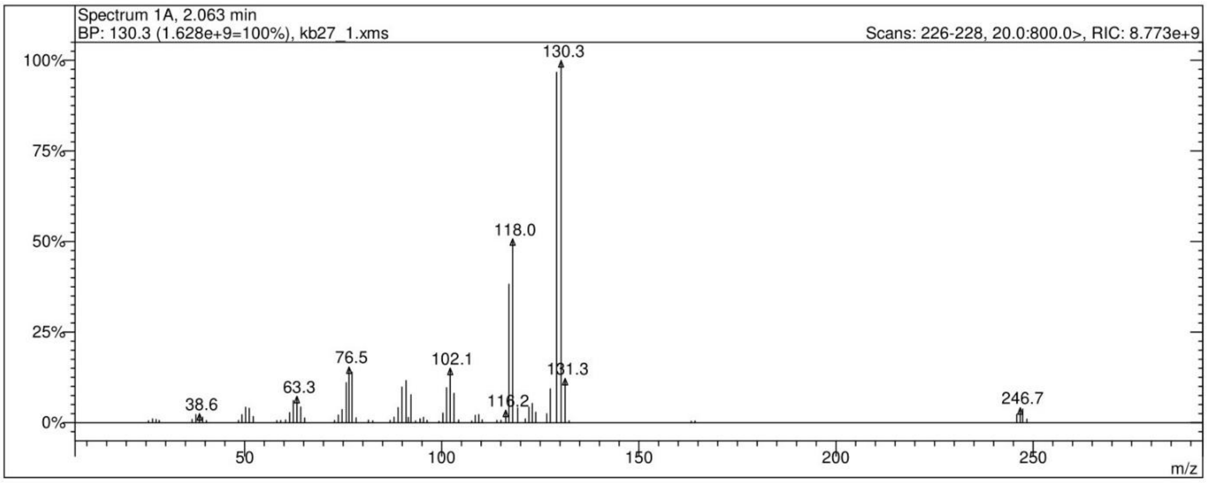


**Figure S20.** EI-MS spectrum of compound **9**

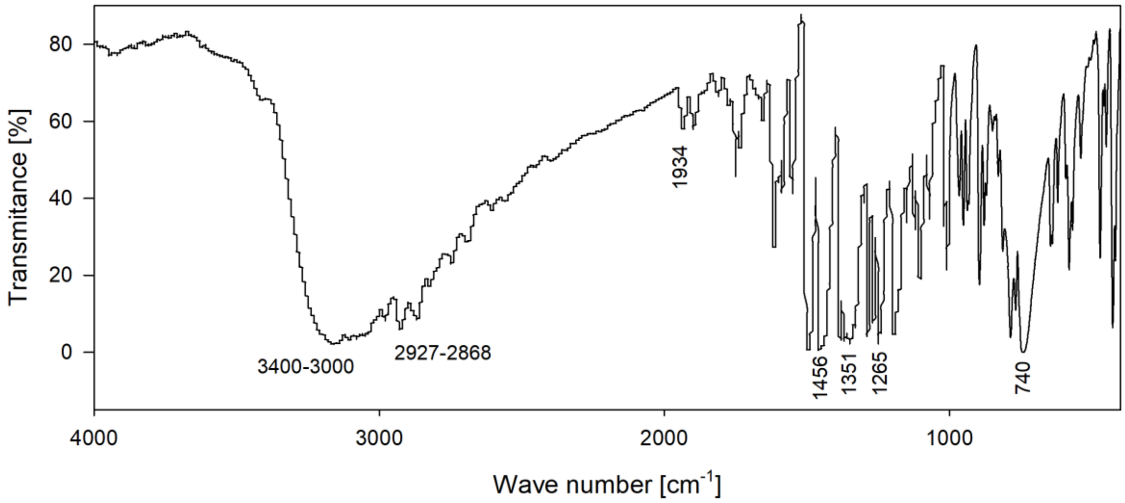


**Figure S21.** IR spectrum of compound **9**

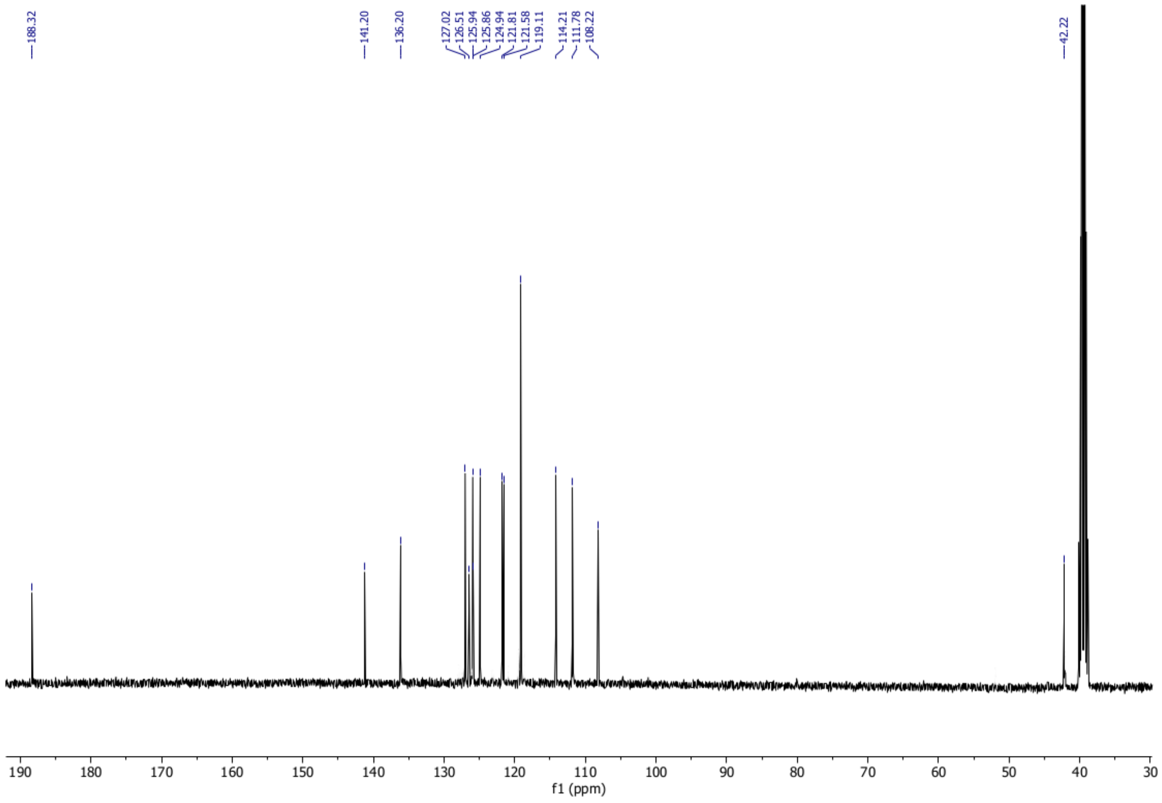


**Figure S22a.** $^{13}$C NMR spectrum of compound **10**

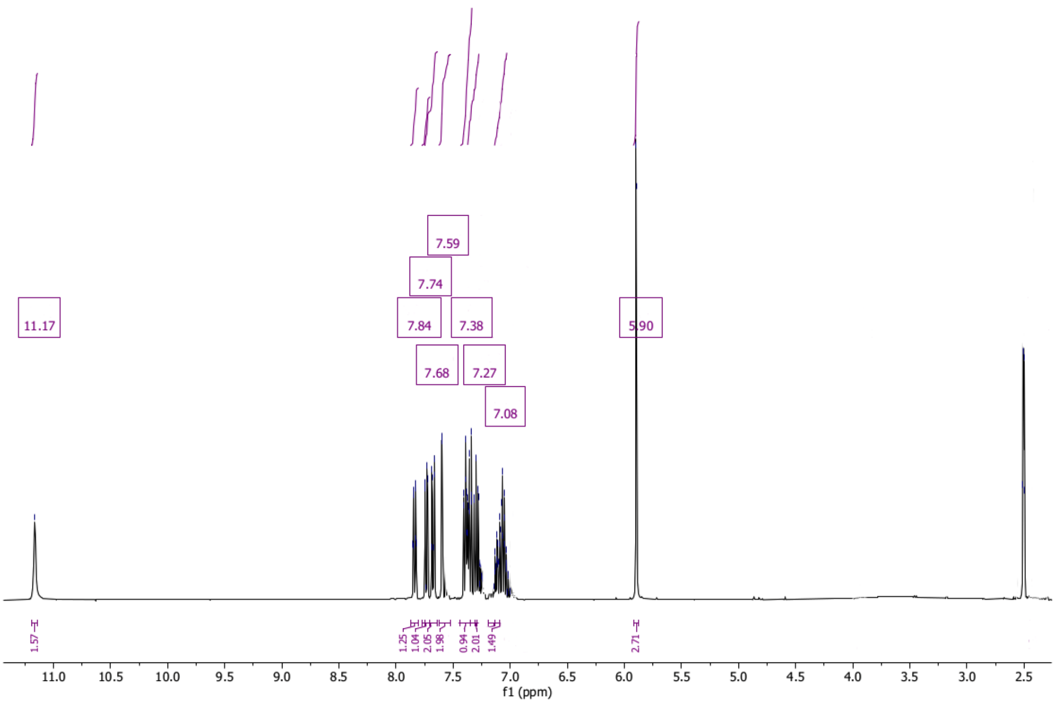


**Figure S22b.** $^{1}$H NMR spectrum of compound **10**

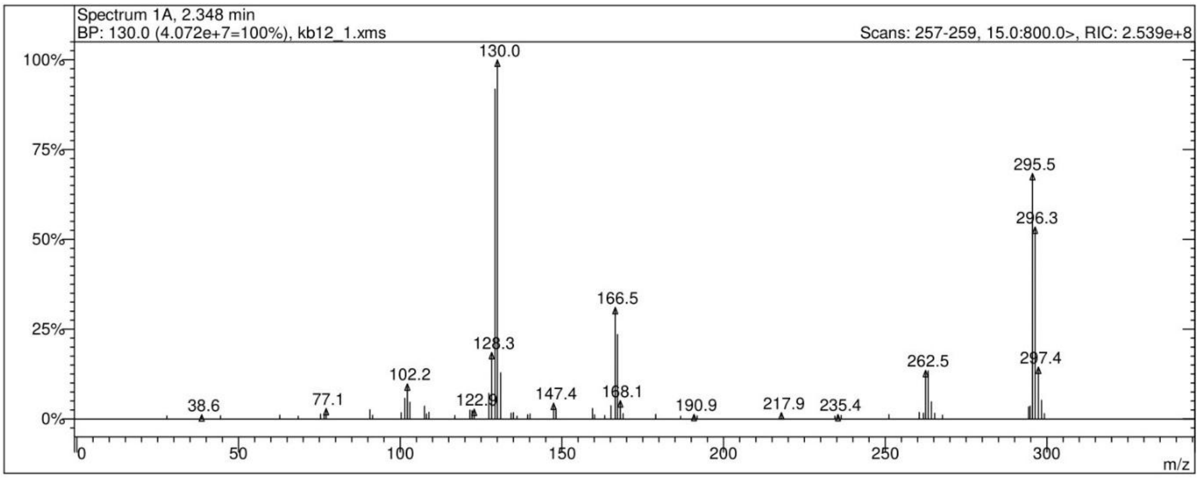


**Figure S23.** EI-MS spectrum of compound **10**

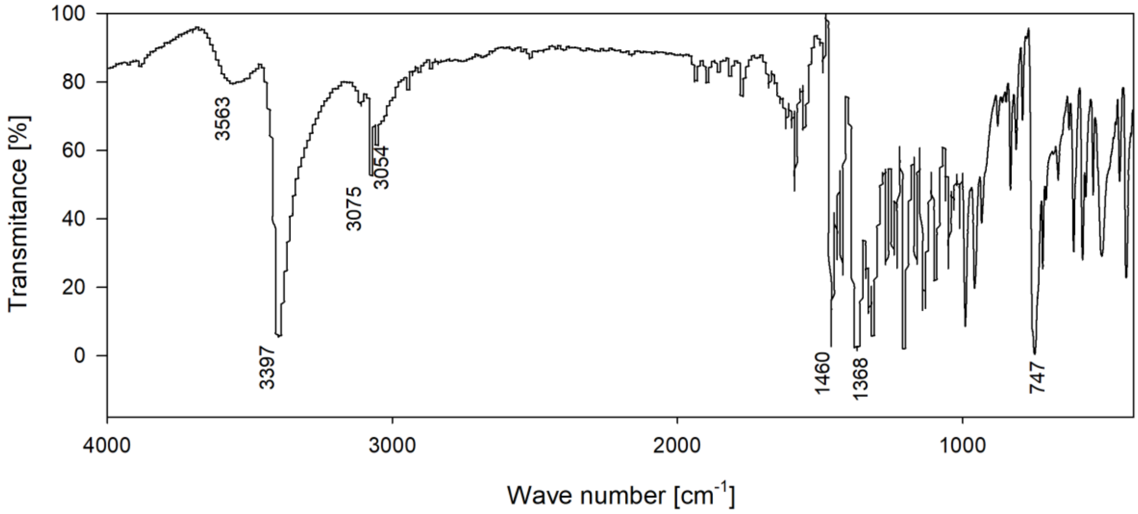


**Figure S24.** IR spectrum of compound **10**

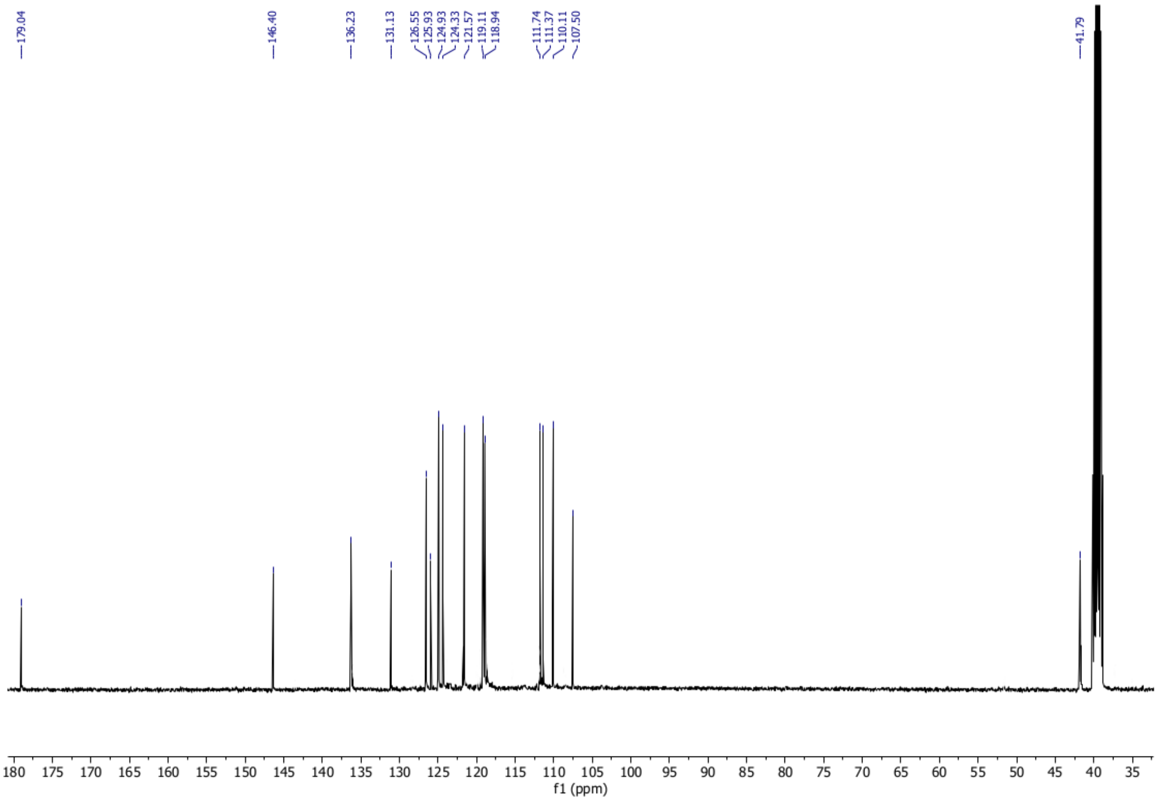


**Figure S25a.** $^{13}$C NMR spectrum of compound **11**

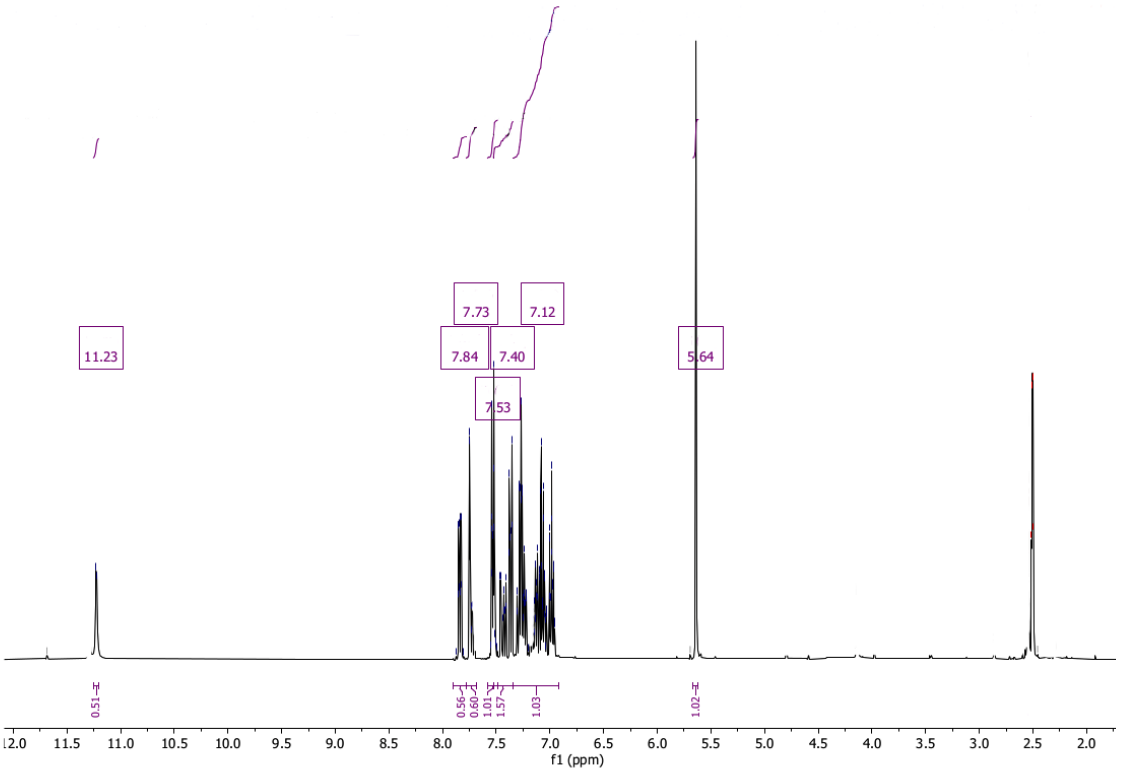


**Figure S25b.** $^{1}$H NMR spectrum of compound **11**

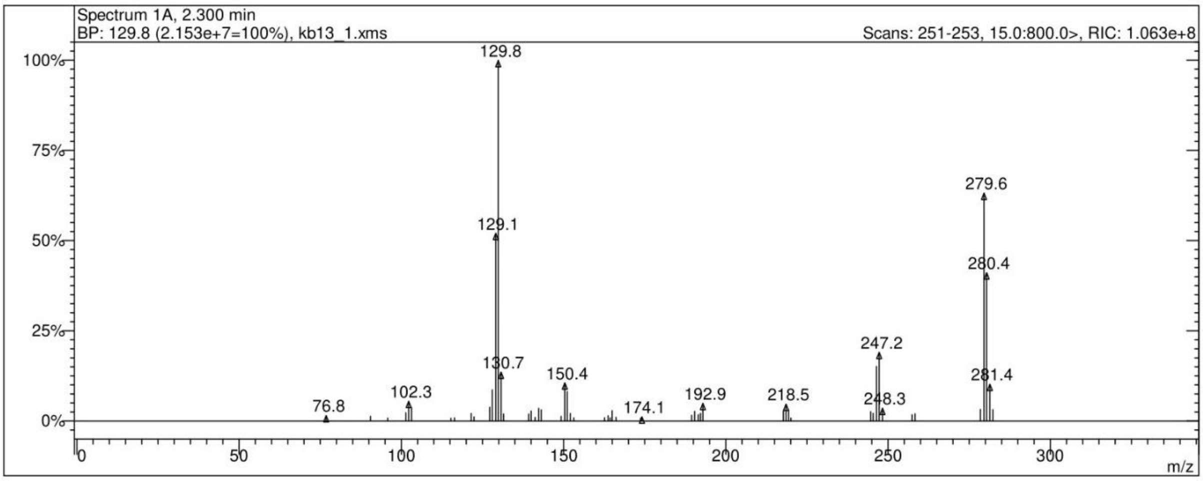


**Figure S26.** EI-MS spectrum of compound **11**

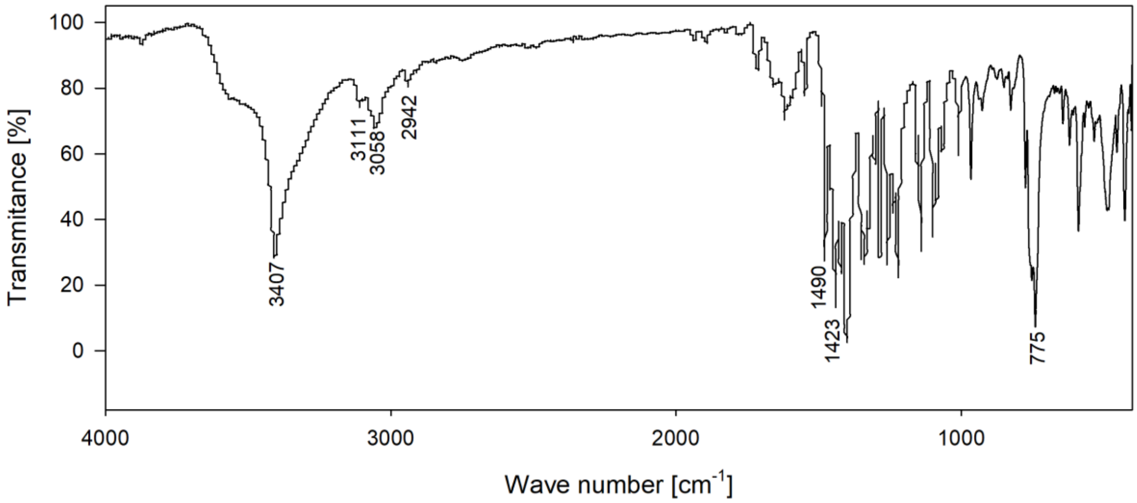


**Figure S27.** IR spectrum of compound **11**

**Table S1**. Hydrogen bond parameters.

| *D*—H···*A* | *D*—H (Å) | H···*A* (Å) | *D*···*A* (Å) | *D*—H···*A* (°) |
|---|---|---|---|---|
| **4** | | | | |
| N1—H1···N3$^{i}$ | 0.86 | 2.07 | 2.905 (2) | 163 |
| N21—H21···N23$^{ii}$ | 0.86 | 2.10 | 2.915 (2) | 157 |
| **5** | | | | |
| N1—H1···N3$^{iii}$ | 0.86 | 2.13 | 2.953 (1) | 159 |
| **8** | | | | |
| N1—H1···N3$^{iv}$ | 0.86 | 2.10 | 2.957 (1) | 173 |
| **10** | | | | |
| N1—H1···$C_g$(C7-C8)$^{v}$ | 0.86 | 2.41 | 3.227 | 158 |
| **11** | | | | |
| N1—H1···S1$^{vi}$ | 0.86 | 2.78 | 3.485 (2) | 140 |

Symmetry code(s): (i) *x*-1, *y*, *z*; (ii) *x*+1, *y*, *z*; (iii) *x*, -*y*+3/2, *z*-1/2; (iv) *x*+1/2, -*y*+3/2, *z*+1/2; (v) *x*-1/2, *y*+1/2, -*z*+1; (vi) *x*, -*y*+1/2, *z*-1/2.

**Table S2.** Crystal data and structure refinement parameters for selected gramine derivatives.

| | **4** | **5** | **8** | **10** | **11** |
|---|---|---|---|---|---|
| Chemical formula | $C_{14}H_{15}N_3$ | $C_{15}H_{17}N_3$ | $C_{18}H_{15}N_3$ | $C_{16}H_{12}N_2S_2$ | $C_{16}H_{12}N_2OS$ |
| $M_r$ | 225.29 | 239.31 | 273.33 | 296.40 | 280.34 |
| Crystal system, space group | Monoclinic, $P2_1/c$ | Orthorhombic, *Pbca* | Monoclinic, $P2_1/n$ | Orthorhombic, $P2_12_12_1$ | Monoclinic, $P2_1/c$ |
| *a, b, c* (Å) | 9.5627 (2), 17.1298 (3), 15.3143 (4) | 10.4215 (1), 13.5948 (2), 18.8911 (2) | 14.0170 (1), 7.0099 (1), 15.0277 (1) | 5.4548 (2), 13.9861 (3), 18.2075 (4) | 13.9368 (1), 7.2337 (1), 13.7132 (1) |
| α, β, γ (°) | 90, 105.097 (2), 90 | 90, 90, 90 | 90, 100.994 (1), 90 | 90, 90, 90 | 90, 100.242 (1), 90 |
| $V$ (Å$^3$) | 2422.01 (9) | 2676.46 (5) | 1449.49 (3) | 1389.08 (7) | 1360.46 (2) |
| $Z$ | 8 | 8 | 4 | 4 | 4 |
| $D_x$ (Mg m$^{-3}$) | 1.236 | 1.188 | 1.253 | 1.417 | 1.369 |
| Radiation type | Cu *K*α | Cu *K*α | Cu *K*α | Mo *K*α | Cu *K*α |
| μ (mm$^{-1}$) | 0.59 | 0.56 | 0.59 | 0.37 | 2.08 |
| Crystal size (mm) | 0.60 × 0.08 × 0.07 | 0.60 × 0.25 × 0.15 | 0.30 × 0.20 × 0.05 | 0.60 × 0.10 × 0.08 | 0.60 × 0.20 × 0.08 |
| Data collection | | | | | |
| $T_{min}$, $T_{max}$ | 0.727, 1.000 | 0.553, 1.000 | 0.792, 1.000 | 0.926, 1.000 | 0.424, 1.000 |
| No. of measured, independent and observed [$I > 2s(I)$] reflections | 39931, 5059, 4028 | 25903, 2798, 2439 | 24636, 3027, 2682 | 36290, 3350, 2871 | 33085, 2857, 2628 |
| $R_{int}$ | 0.054 | 0.033 | 0.026 | 0.039 | 0.044 |
| (sin θ/λ)$_{max}$ (Å$^{-1}$) | 0.632 | 0.631 | 0.630 | 0.671 | 0.630 |
| Refinement | | | | | |
| $R[F^2 > 2s(F^2)]$, $wR(F^2)$, $S$ | 0.071, 0.239, 1.08 | 0.042, 0.128, 1.06 | 0.038, 0.108, 1.06 | 0.044, 0.083, 1.15 | 0.042, 0.131, 1.10 |
| No. of reflections | 5059 | 2798 | 3027 | 3350 | 2857 |
| No. of parameters | 318 | 165 | 191 | 181 | 182 |
| No. of restraints | 26 | 0 | 0 | 0 | 12 |
| $\Delta\rho_{max}$, $\Delta\rho_{min}$ (e Å$^{-3}$) | 0.51, -0.51 | 0.11, -0.18 | 0.13, -0.16 | 0.19, -0.18 | 0.20, -0.32 |
| Absolute structure parameter | – | – | – | 0.00 (2) | – |
